# Targeting of the CD80/86 proinflammatory axis as a therapeutic strategy to prevent severe COVID-19

**DOI:** 10.1038/s41598-021-90797-0

**Published:** 2021-06-01

**Authors:** Antonio Julià, Irene Bonafonte-Pardàs, Antonio Gómez, María López-Lasanta, Mireia López-Corbeto, Sergio H. Martínez-Mateu, Jordi Lladós, Iván Rodríguez-Nunez, Richard M. Myers, Sara Marsal

**Affiliations:** 1 Rheumatology Department and Rheumatology Research Group, Vall d’Hebron Hospital Research Institute, 08035 Barcelona, Spain; 2 HudsonAlpha Institute for Biotechnology, Huntsville, AL USA

**Keywords:** Target identification, Viral infection, Inflammatory diseases

## Abstract

An excessive immune response known as cytokine storm is the hallmark of severe COVID-19. The cause of this cytokine rampage is yet not known. Based on recent epidemiological evidence, we hypothesized that CD80/86 signaling is essential for this hyperinflammation, and that blocking this proinflammatory axis could be an effective therapeutic approach to protect against severe COVID-19. Here we provide exploratory evidence that abatacept, a drug that blocks CD80/86 co-stimulation, produces changes at the systemic level that are highly antagonistic of the proinflammatory processes elicited by COVID-19. Using RNA-seq from blood samples from a longitudinal cohort of n = 38 rheumatic patients treated with abatacept, we determined the immunological processes that are significantly regulated by this treatment. We then analyzed available blood RNA-seq from two COVID19 patient cohorts, a very early cohort from the epicenter of the pandemic in China (n = 3 COVID-19 cases and n = 3 controls), and a recent and larger cohort from the USA (n = 49 severe and n = 51 mild COVD-19 patients). We found a highly significant antagonism between SARS-CoV-2 infection and COVID-19 severity with the systemic response to abatacept. Analysis of previous single-cell RNA-seq data from bronchoalveolar lavage fluid from mild and severe COVID-19 patients and controls, reinforce the implication of the CD80/86 proinflammatory axis. Our functional results further support abatacept as a candidate therapeutic approach to prevent severe COVID-19.

## Introduction

SARS-CoV-2 is the seventh coronavirus known to infect humans. Infection with SARS-CoV-2 virus can lead to different degrees of symptomatology and severity, ranging from asymptomatic to an extreme immune response leading to patient death^1^. The fatality rate of COVID-19 is estimated to be close to 1%, which is 10 times more than typical seasonal influenza^2^. COVID-19 has been associated to the cytokine storm or cytokine release syndrome (CRS)^3^, an overload of proinflammatory cytokines that can lead to massive organ failure. This uncontrollable inflammatory process principally occurs in the lungs and, despite the help of mechanical ventilation, it leads to organ collapse and death in many patients^4^. Consequently, there is a major need to identify therapies that can prevent COVID-19 patients from progressing into the life-threatening hyperinflammatory stage^5^.

Soon after the first incidence peak of COVID-19 in Spain in April 2020, we provided evidence^6^ suggesting that anti-interleukin 6 (IL6) therapy (tocilizumab) and blocking of the CD80/86 axis with CTLA4-Ig (abatacept) are associated with a lower prevalence of COVID-19-associated symptomatology in rheumatic patients. The former observation was in line with the importance of IL6 in severe COVID-19 and the cytokine storm provided by the first clinical studies of COVID-19 patients^7^. Based on this preliminary evidence, drugs that target this cytokine have been used off-label to attempt to rescue critically ill patients^8^, and there is currently a large number of clinical trials evaluating its utility. However, when this study was conducted, blocking of the proinflammatory CD80/86 axis with abatacept had not been yet considered as a candidate therapeutic approach to prevent severe COVID-19.

Abatacept is a fusion protein consisting of the extracellular domain of human cytotoxic T lymphocyte antigen 4 (CTLA4) linked to the modified Fc domain of human IgG1^9^. It binds to both costimulatory proteins CD80 and CD86 on professional antigen presenting cells (dendritic cells, macrophages and B cells) with higher affinity than CD28 on the surface of T cells, thereby preventing the necessary costimulatory signal required by T cells to progress to activation. It was originally developed to reduce inflammation in rheumatoid arthritis (RA), where T cell activation is central to the disease pathology, and has been recently approved also for the treatment of psoriatic arthritis and juvenile idiopathic arthritis^9^.

In severe COVID-19, macrophages in the lungs activate and produce large amounts of proinflammatory cytokines, mainly IL6. Recent evidence suggests that during SARS-CoV-2 infection, resident alveolar macrophages are gradually substituted by monocyte-derived proinflammatory macrophages^10,11^. However, how these macrophages are brought to drastically increase the proinflammatory cytokine production in severe COVID-19 is yet not known^5^. An increase in proinflammatory signals by hyperactivated T cells is a possible explanation^12^. Co-stimulation through the CD80/86 axis is a key mechanism for T-cell activation. We hypothesize that blocking this co-stimulation with abatacept could be an effective way to dampen the excessive T cell hyperactivation and subsequent macrophage cytokine rampage that leads to severe COVID-19. This mechanism could explain the lower prevalence of COVID-19 symptomatology observed in abatacept-treated patients. Here we provide additional evidence supporting the utility of blocking the CD80/86 axis to prevent COVID-19 associated hyperinflammation. Using RNA-seq, we have characterized the longitudinal response to abatacept at the systemic level. Analyzing blood RNA-seq data from two different COVID-19 cohorts, we demonstrate that this therapeutic response is highly antagonistic to that elicited by COVID-19. Our results provide additional evidence at the functional level that blocking the CD80/86 axis could be a useful therapeutic approach to prevent severe COVID-19.

## Results

### CD80/86 blocking antagonizes COVID-19 systemic features

**Figure 1:**
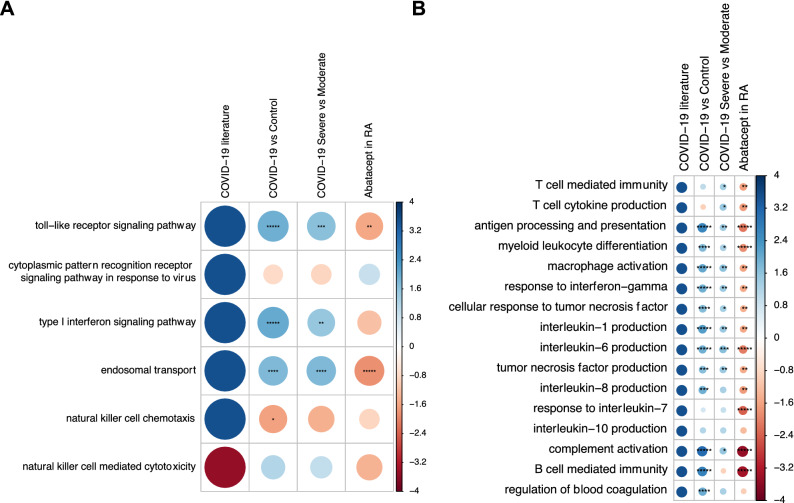
Changes induced by abatacept in key COVID-19 pathological processes. (**A**) Biological processes associated with immune viral response (first step of COVID-19 response). (**B**) Biological processes associated with hyperinflammation and severity (second step in COVID-19). The first column indicates the direction of change of the biological process induced by COVID19 as described in different clinical studies (solid blue: up-regulation, solid red: down-regulation, as measured by the GSEA Normalized Enrichment Score -NES-). The last column shows the direction (blue: upregulation, red: downregulation) and statistical significance of the change induced in the COVID-19 associated processes at the systemic level by the treatment with abatacept. Circle size is also proportional to the NES. P-value significance levels are indicated as *< 0.05, **< 0.005, ***< 0.0005, ****< 0.00005 and *****< 0.000005. The two middle columns show the change and statistical significance of these processes in the early COVID-19 cohort (comparing COVID-19 patients to healthy controls), and the late COVID-19 cohort (comparing severe to mild cases).

In our first analysis, we evaluated how the biological features associated with COVID-19 pathology at the systemic level are affected by treatment with abatacept. A total of 22 processes representing previously described anti-viral and hyperinflammatory responses during COVID-19 were evaluated (Supplementary Table S1). Using a longitudinal cohort of n = 38 rheumatoid arthritis (RA) patients treated with abatacept, we tested whether these COVID-19-related processes are modified during CD80/86 axis blocking. Using this approach, we found that 16 COVID-19 associated processes were significantly modified by treatment with abatacept at the systemic level (Fig. 1A,B, Supplementary Fig. S1A,B, Supplementary Table S2). These pathways include processes crucial for COVID-19 severity including T cell mediated immunity, macrophage activation and cytokine production (IL1, IL6, IL8 and TNF, among others). Importantly, all the functional changes occurred in the opposite direction to that previously described in COVID-19 pathology.

**Figure 2:**
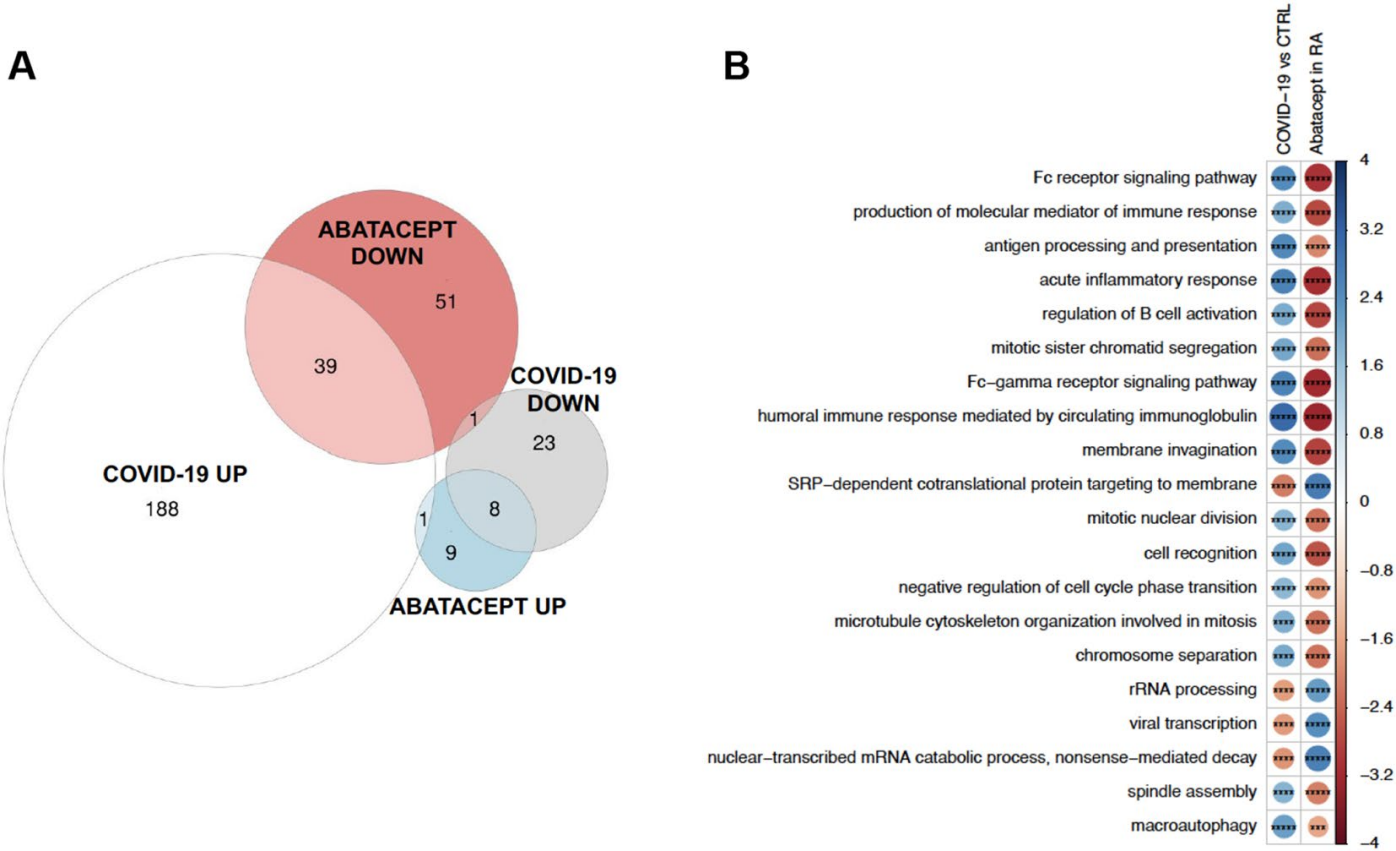
Biological processes modified by COVID-19 and by treatment with abatacept: early cohort. COVID-19 associated processes represent processes dysregulated in COVID-19 patients compared to healthy controls in the early cohort. (**A**) Multiple Venn plot showing the biological processes significantly modified by each exposure (FDR < 0.05) and the resulting disjoint combinations. From the overlapping processes, 47 out of 49 (96%) were found to be modified antagonistically by abatacept while only 2 (4%) processes showed changes in the same direction. (**B**) 20 top biological pathways that show a higher level of antagonism between COVID-19 exposure and treatment with abatacept. FDR-adjusted P-values from the two analyses were combined using Fisher’s method, and the resulting combined P-value are used here as a ranking measure of antagonism. Blue indicates upregulation and red downregulation of the pathway as measured by the GSEA Normalized Enrichment Score, which is also reflected in the circle size. FDR-corrected P-value levels are indicated as *< 0.05, **< 0.005, ***< 0.0005, ****< 0.00005 and *****< 0.000005.

To provide additional evidence supporting the antagonism of abatacept to COVID-19, we directly analyzed the perturbation of the systemic gene expression caused by COVID-19. For this objective, we determined the biological pathways differentially activated by COVID-19 using two different COVID-19 datasets. First, we used RNA-seq data from a very *early cohort* of PBMC data from 3 COVID-19 patients and 3 healthy controls obtained at the epicenter of the pandemic in Wuhan, China^13^. After adjusting for granulocyte percentage, we found that, from a total of 6,187 biological pathways, 260 were differentially activated due to COVID-19 (FDR < 0.05, Supplementary Table S3). The same analysis approach in our longitudinal cohort identified 109 biological pathways significantly associated to the treatment with abatacept (FDR < 0.05, Supplementary Table S4). We next determined the level of overlap between the two exposures and found a total of 49 pathways significantly modified by both COVID-19 and abatacept (Fig. 2A). From these, 47 (96%) were modified by abatacept in an opposite direction to that elicited by COVID-19. The probability that this level of antagonism occurred by chance, is very low (*P* value < 2.5e−18, binomial test). Figure 2B shows the biological pathways that are more antagonistically modified by abatacept in relation to COVID-19. Among the pathways most significantly antagonized, B cell activation and Fc-mediated immune response were found to be highly activated during COVID-19 and significantly repressed by treatment with abatacept. The complete list of overlapping pathways is available in Supplementary Table S5.

**Figure 3:**
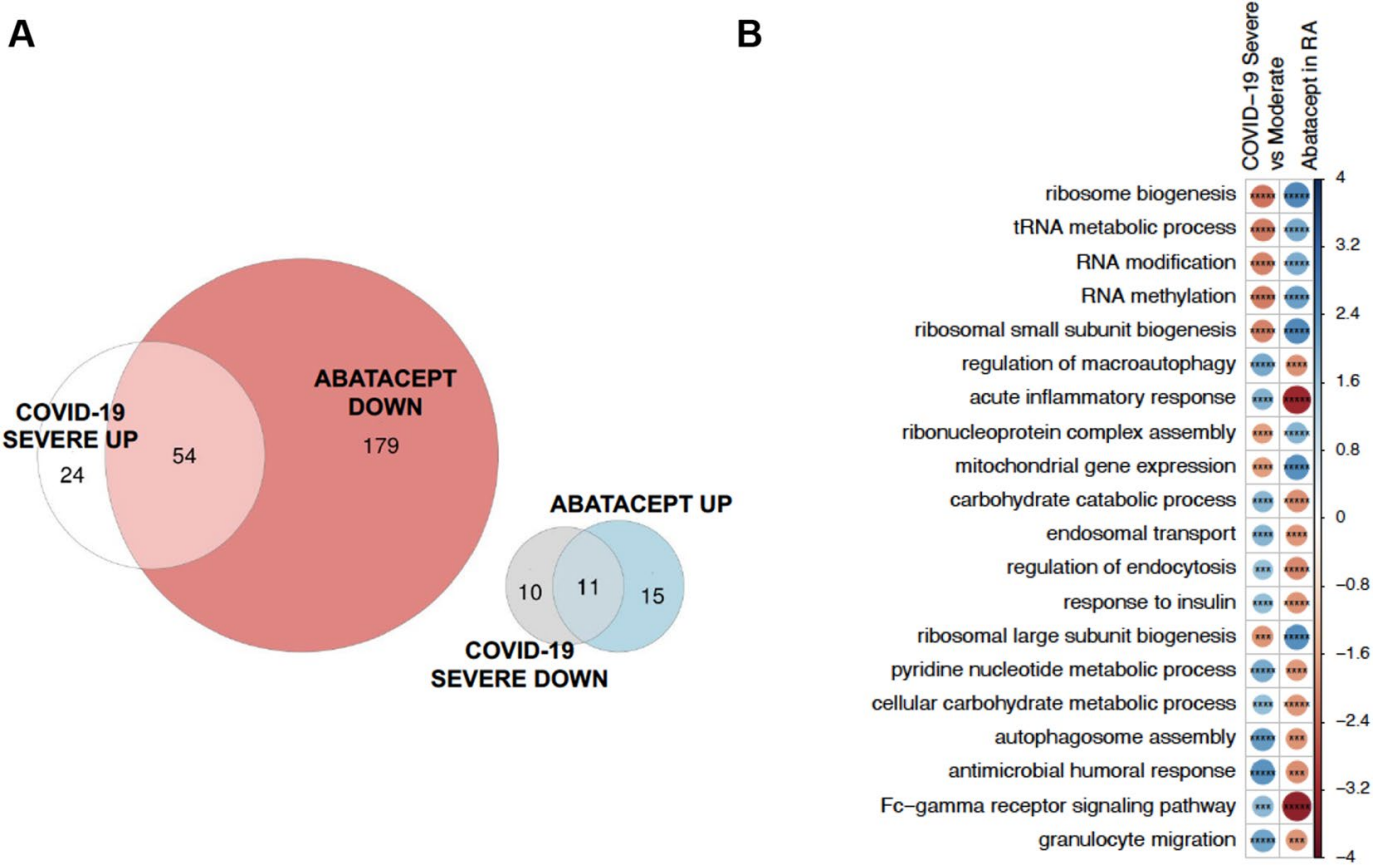
Biological processes distinguishing COVID-19 severe and moderate patients and modified by treatment with abatacept. Severe COVID-19 associated processes represent processes dysregulated in severe COVID-19 patients compared to moderate patients in the late cohort. (**A**) Multiple Venn plot showing the biological processes significantly modified by each exposure (FDR < 0.05) and the resulting disjoint combinations. From the overlapping processes, 100% were found to be modified antagonistically by abatacept. (**B**) Top 20 biological pathways that show a higher level of antagonism between COVID-19 exposure and treatment with abatacept. FDR-adjusted P-values from the two analyses are combined using Fisher’s method, and the resulting combined P-value are used as a ranking measure of antagonism. Blue indicates upregulation and red downregulation of the pathway as measured by the GSEA Normalized Enrichment Score, which is also reflected in the circle size. FDR-corrected P-value levels are indicated as *< 0.05, **< 0.005, ***< 0.0005, ****< 0.00005 and *****< 0.000005.

In October 2020, a larger dataset on COVID-19 patients became available (*late cohort*), consisting on a transcriptomic, metabolomic, lipidomic and proteomic data from moderate and severe COVID-19-positive patients as well as COVID-negative patients admitted to the hospital^14^. The whole blood RNA-seq data from n = 51 moderate COVID-19 patients and n = 49 severe patients from this study allowed us not only to corroborate our findings in the early cohort, but also to specifically test for association with clinical severity. From 6187 tested biological pathways, a total of 99 pathways were significantly enriched when comparing severe to moderate COVID-19 patients (FDR < 0.05, Supplementary Table S6). Using the same approach, 259 biological pathways were associated with the response to abatacept in our cohort (FDR < 0.05, Supplementary Table S7). Comparing the two exposures, we found that 67 pathways were significantly associated with both COVID-19 severity and the response to therapy (Fig. 3A). Importantly, all of these common pathways were modified by abatacept in the opposite direction to that of severe COVID-19. This level of antagonism was highly unlikely to occur by chance (*P* value < 3.07e−35). Figure 3B shows the biological pathways that are more antagonistically modified by abatacept in relation to severe COVID-19. Key biological processes associated with severity, like platelet activation and interleukin-6 production, were among the most significantly antagonized biological pathways. The complete list of overlapping pathways is available in Supplementary Table S8.

At the single-gene level, from the 44 genes differentially expressed in response to Abatacept therapy (FDR-adjusted P-value < 0.05), 15 were found to be expressed also in the COVID-19 late cohort. From these, five genes were differentially expressed in severe COVID-19 patients compared to mild patients (P-value < 0.05, Supplementary Table S9). Importantly, all five genes—*HJURP*, *GTSE1*, *TYMS*, *CDC20* and *BIRC5*- are down-regulated by abatacept and up-regulated in severe COVID-19 patients.

### Survival analysis: antagonism to abatacept signature as a predictor of severity

**Figure 4:**
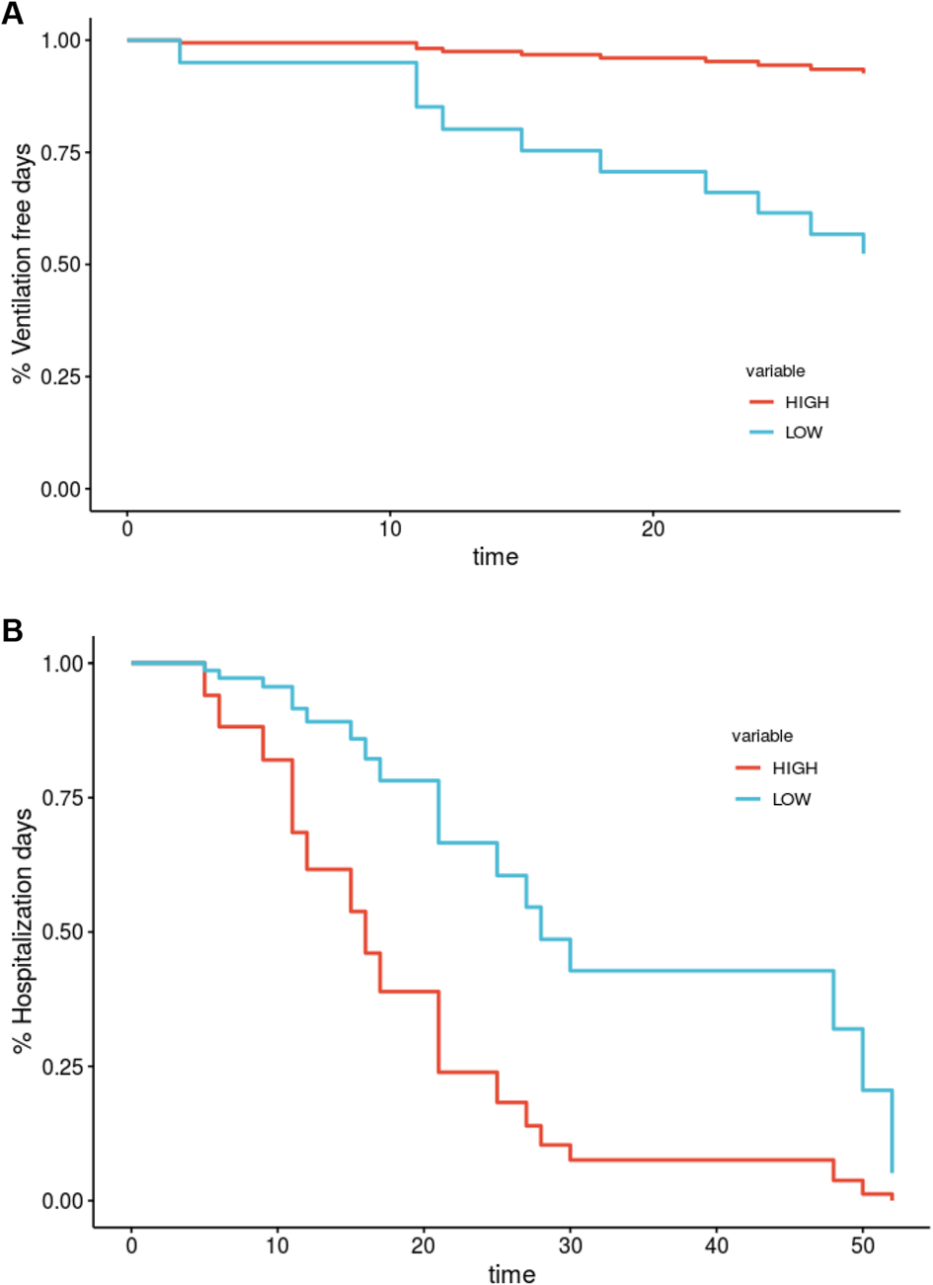
Kaplan–Meier analyses of severity outcomes on COVID-19 patients admitted to the hospital. (**A**) Kaplan–Meier curve for the analysis of the time to mechanical ventilation during a 28-day time window. Patients with a gene expression profile more similar to abatacept (ABS-High) had a lower probability to require mechanical ventilation (*P* = 0.047). (**B**) Kaplan–Meier curve for the analysis of hospitalization days. COVID-19 patients with a gene expression profile more different to abatacept (ABS-Low) had a higher probability to spend more days in the hospital (*P* = 0.032).

To further corroborate the antagonism of the abatacept response signature to COVID-19, we explored its utility as a biomarker to predict severity. For this objective, we built a score (abatacept score, ABS) using the genes differentially expressed by treatment with abatacept (n = 15, Supplementary Information). This set was enriched in immunoglobulin encoding genes, suggesting a predominant gene expression change in the B cell compartment. Using this gene expression signature, we identified the COVID-19 patients in the late cohort with more similar (ABS-High) and dissimilar (ABS-Low) expression. A total of 11 COVID-19 patients were classified as ABS-High and 20 were classified as ABS-Low. Using this patient categorization, survival analyses were performed using two critical and complementary measures of severity: number of days without mechanical ventilation and number of days in the hospital. In both cases, we found that ABS-High patients (i.e. patients with a gene expression profile more similar to the abatacept-response signature) were significantly associated with a better prognosis (Fig. 4A,B, Supplementary Fig. S2A,B). ABS-Low patients, instead, had a higher probability to require mechanical ventilation (P = 0.047, log-rank test) and to spend more days in the hospital (P = 0.032, log-rank test).

### Association of CD80/86 axis elements in COVID-19

**Figure 5:**
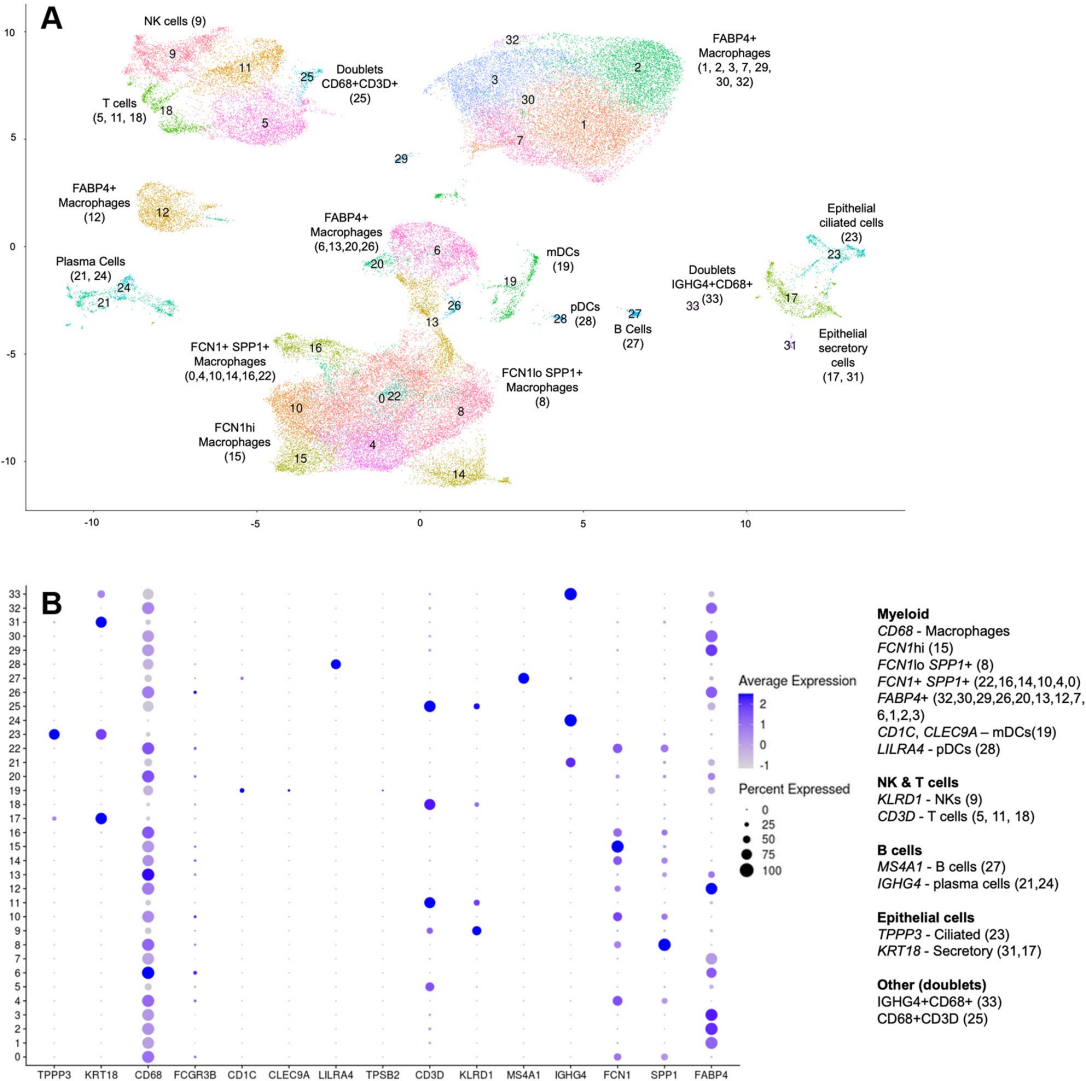
(**A**) UMAP plot of the single cell RNA-seq clustering from the bronchoalveolar samples from COVID-19 and healthy control individuals. A total of 34 clusters were found in this sample of 6 severe and 3 mild COVID-19 patients as well as 3 healthy controls. Each cluster is colored differently and depicted by a number. (**B**) Expression of marker genes across clusters. The markers are used to identify the cell types constituting each cluster, as indicated on the right. Circle size is proportional to the percentage of cells in each cluster expressing the marker and circle color represents average marker gene expression in the cluster.

**Figure 6:**
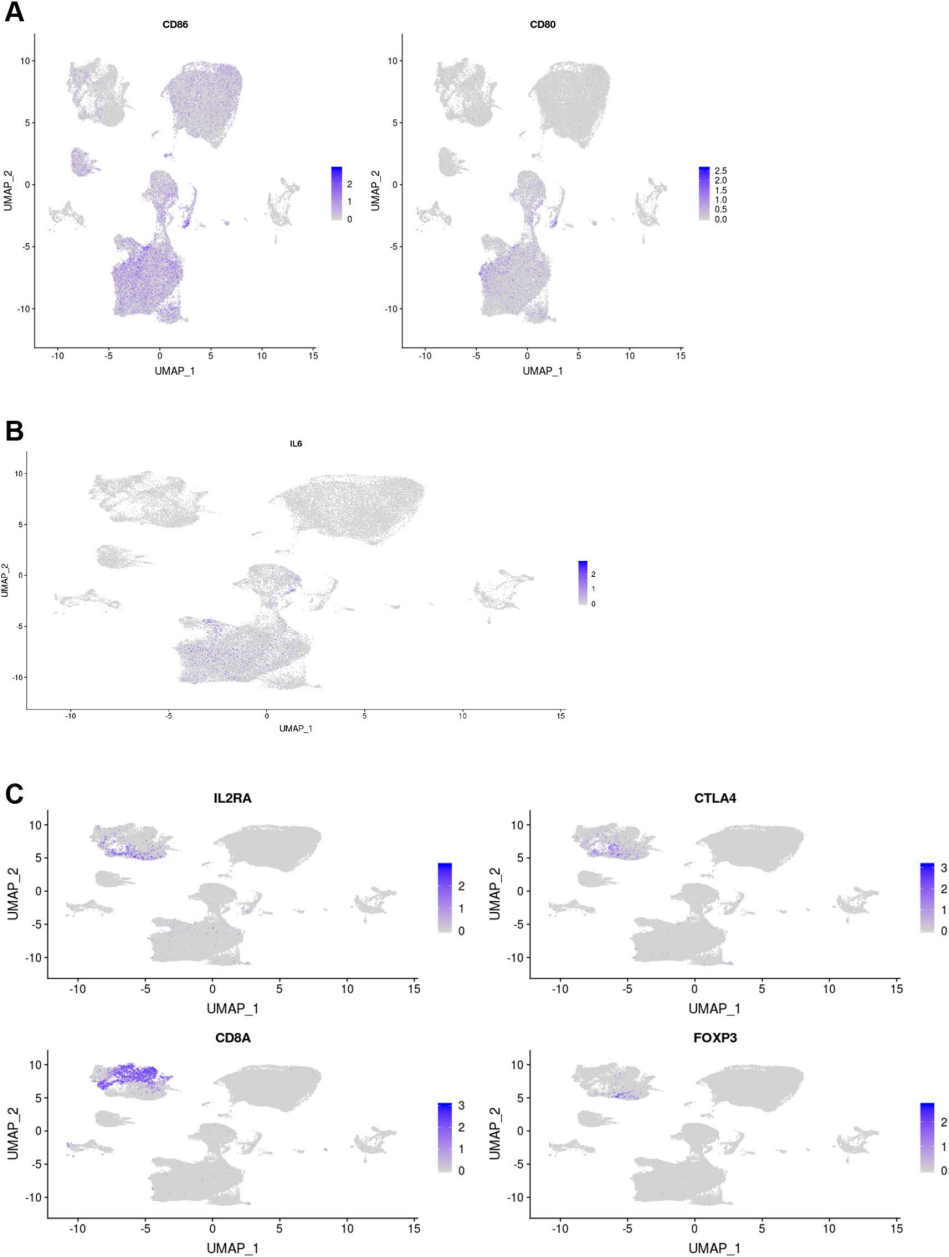
Expression of the cell-type specific markers defining the cell clusters representing the three elements of the CD80/86 axis. (**A**) Expression of co-stimulatory protein genes CD80 and CD86, and targets of abatacept. While CD86 has a more ubiquitous expression (including both alveolar and tissue infiltrating macrophages), CD80 is specifically expressed in FCN1+ monocyte-derived macrophages. (**B**) IL6 expression is circumscribed to the clusters of activated macrophages. (**C**) Cell markers representing activated T cells (CTLA4, IL2RA), CD8+ lymphocytes (CD8A) and Tregs (FOXP3 and IL2RA). CTLA4 expression was enriched in the CD4+ T cell cluster number 5.

After identifying a significant antagonism between the response to abatacept and COVID-19 pathology at the systemic level, we next sought to corroborate the relevance of this immune-signaling axis in the target tissue of COVID-19 severity. While COVID-19 can affect multiple organs, the lungs are the predominant target of the severity of the immune response and where the cytokine storm originates^3^. To evaluate the relevance of CD80/86 co-stimulation in the lungs during COVID-19 we took advantage of an available single-cell RNA-seq dataset generated from the bronchoalveolar lavage fluid obtained from mild (n = 3) and severe (n = 6) COVID-19 patients and from healthy controls (n = 3)^10,11^. Figure 5A,B show the cell clustering analysis and identification of the marker genes within each cell type, respectively. Based on this clustering, Fig. 6A–C display the expression of gene markers of the key elements of the C80/86 activation axis, respectively.

**Figure 7:**
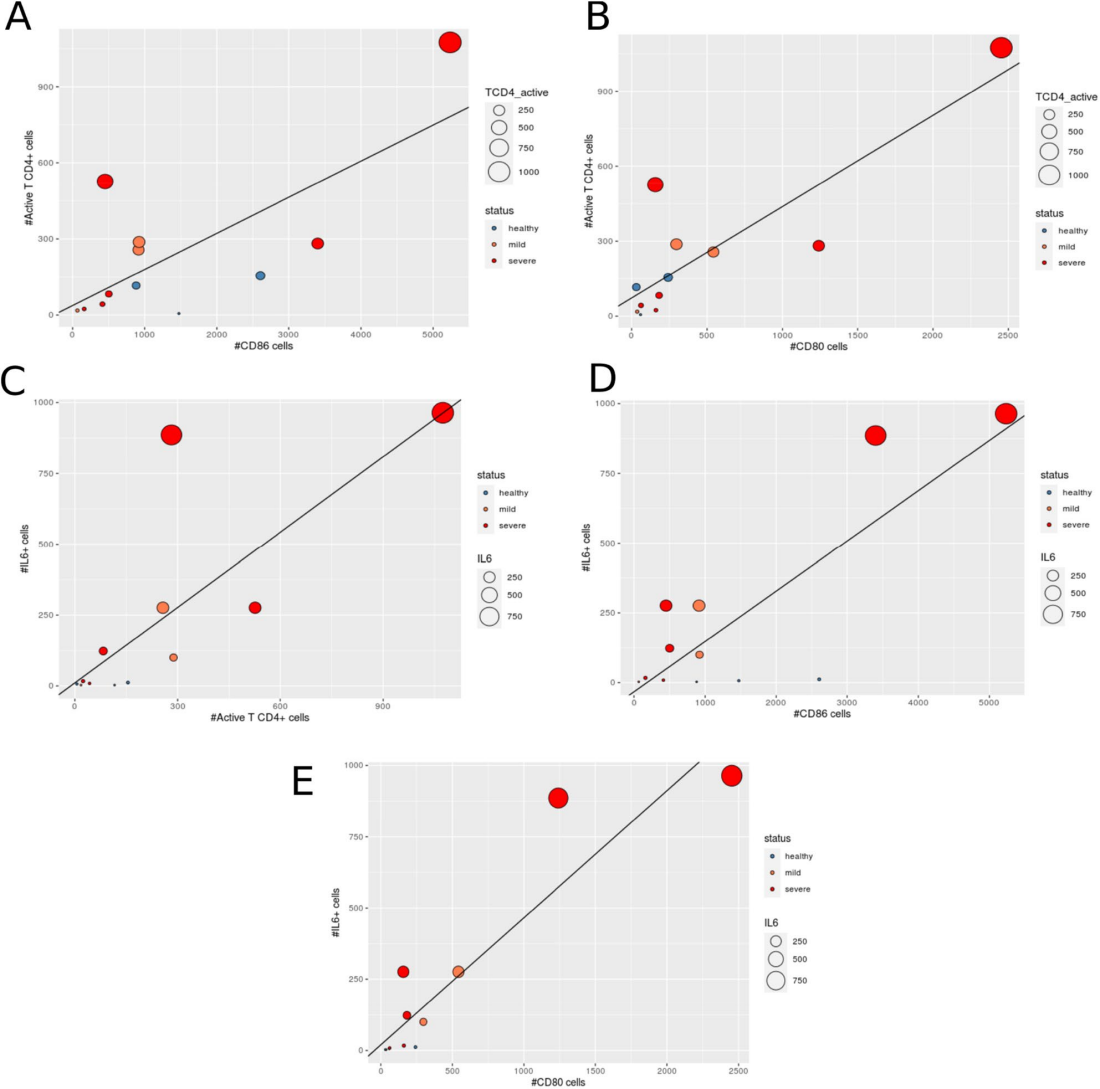
Elements of the CD80/86 axis in bronchoalveolar lavage fluid cells obtained from COVID-19 patients and controls. Plots showing the relationship between the elements of the proinflammatory signaling axis targeted by abatacept: CD80^+^ or CD86^+^ APCs, active CD4+ T cells and IL-6 producing macrophages. (**A**) CD86^+^ cells vs active CD4+ T cells (P = 0.013). (**B**) CD80^+^ cells vs active CD4+ T cells (P = 0.034). (**C**) active CD4+ T cells vs IL6^+^ cells (P = 0.067). (**D**) CD86^+^ cells vs IL6^+^ cells (P = 0.00036). (**E**) CD80^+^ cells vs IL6^+^ cells (P = 0.15). Gene expression units represent the corrected counts after applying normalization and variance stabilization approach implemented in *scTransform*.

According to our hypothesis, an excess of CD80/86 co-stimulation of antigen-presenting cells (APCs) to CD4+ T cells in SARS-CoV-2 infected individuals leads to a hyperactivation of macrophages that subsequently ramp up their production proinflammatory cytokines, mainly IL6. Treatment with abatacept would dampen this signaling axis, thereby reducing the likelihood of developing a cytokine storm. In this disease severity model, we would expect to find an association between the abundance of the three key elements of the CD80/86 axis: CD80/86^+^ APCs, activated CD4+ T cells and IL-6 producing macrophages. Using the single-cell BALF dataset, we found a significant and positive association between several of the pairwise combinations of the three elements of the CD80/86 axis (Fig. 7A–E): (i) CD80^+^ and CD86^+^ APCs vs active CD4+ T cells (β (95% CI) 0.52 (0.04–0.99), P = 0.034; and β (95% CI) 0.12 (− 0.01 to 0.24), P = 0.067, respectively) (Fig. 7A,B), (ii) activated CD4+ T cells vs IL6-producing macrophages (β (95% CI) = 1.01 (0.26–1.74), P = 0.013) (Fig. 7C) and (iii) CD80^+^ APCs vs IL6-producing macrophages (β (95% CI), P = 0.00036) (Fig. 7D). Conversely, there was no association between other pairs of cell types not specifically involved in this axis, like active T cells numbers and epithelial ciliated cells (cluster #23, P-value = 0.54) or plasma cells (cluster #21) and macrophages (P-value = 0.49).

## Discussion

Since the beginning of the pandemic, SARS-CoV-2 has infected more than 67 million people and caused more than 1,544,000 deaths world-wide as of December 8 2020 (https://coronavirus.jhu.edu/map.html). There is a pressing need to identify therapies that can prevent COVID-19 patients from reaching the hyperinflammatory life-threatening stage known as cytokine storm. Based on previous epidemiological evidence from rheumatic patients treated with several targeted immunomodulators, we hypothesized that blocking of the CD80/86 proinflammatory axis could be a useful therapeutic approach to prevent severe COVID-19. Here we provide additional functional evidence to support this hypothesis. To do so, we have characterized the systemic response to abatacept in a longitudinal cohort of rheumatic patients and subsequently tested for its antagonism to COVID-19 pathological features. Using two patient cohorts, we have found a high level of antagonism of abatacept-mediated response to COVID-19 pathology. The results of this study support abatacept as a candidate therapy to prevent severe COVID-19.

**Figure 8:**
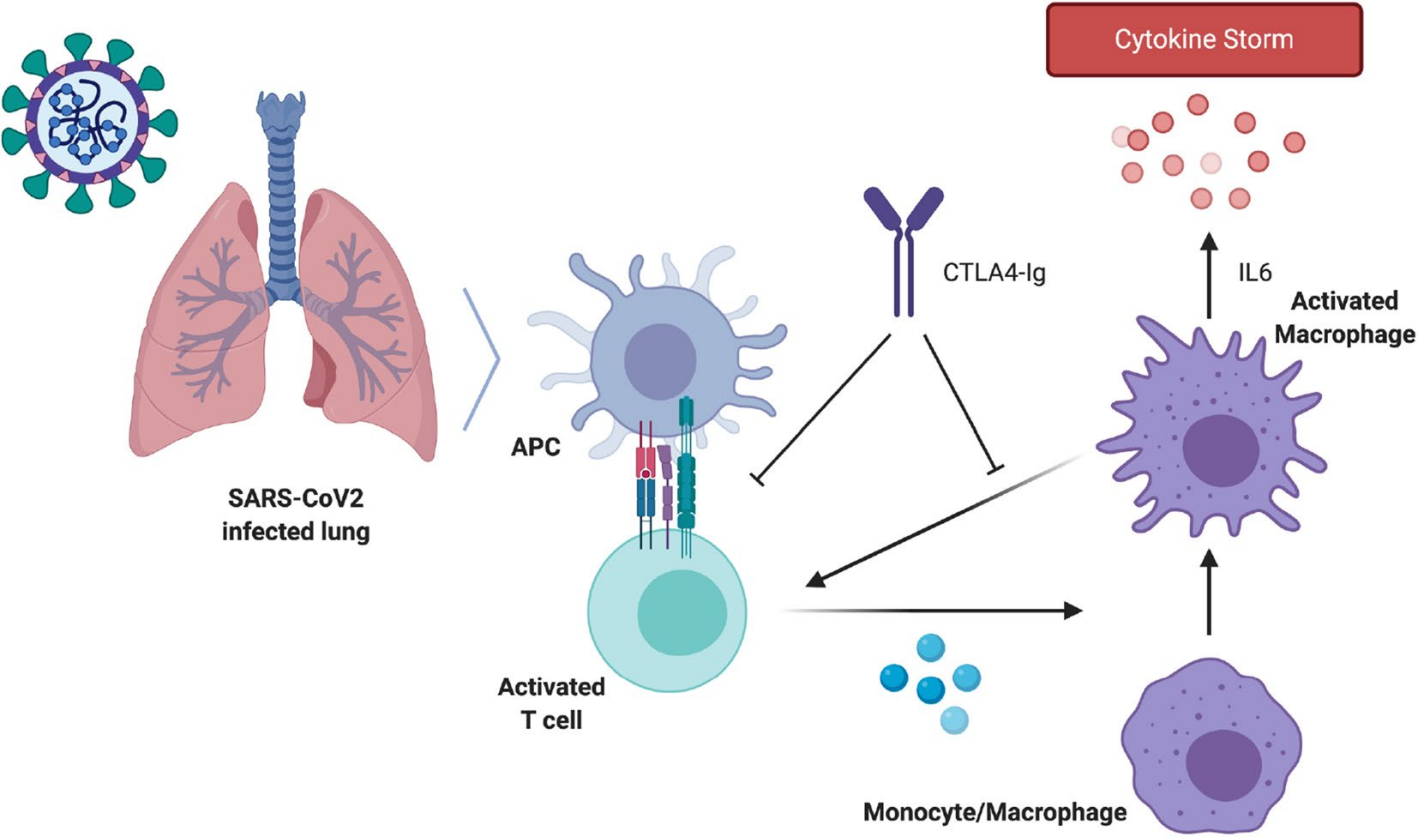
Abatacept dampening of T cell co-stimulation and excessive cytokine production in COVID-19. Abatacept is a recombinant CTLA4Ig fusion protein that selectively blocks CD80 or CD86 co-stimulation of T cells by antigen presenting cells (APCs). Reducing the level of CD4+ T cell activation with abatacept could be a useful therapeutic strategy to prevent reaching the hyperinflammatory state that defines severe COVID-19. Once an individual becomes infected with SARS-CoV-2, an immune response is mounted (first stage of COVID-19). In some individuals, inadequate clearance of the coronavirus by the anti-viral immune response leads to a progressive hyperinflammation (second stage of COVID-19). In this stage, CD80/86-expressing APCs in the lungs-alveolar and monocyte-derived macrophages-activate CD4+ T cells which, in turn, stimulate macrophages to produce large quantities of proinflammatory cytokines, predominantly IL6. The large pool of CD80/86^+^ APCs in the lungs together with the rapidly spreading capacity of SARS-CoV-2 produce a dangerous combination that can lead to an abrupt and life-threatening overexpression of proinflammatory cytokines known as the cytokine storm. Blocking of the CD80/86^+^ axis with abatacept would reduce the probability of reaching this hyperinflammatory stage and therefore prevent severe COVID-19.

Our results suggest that T cell stimulation by CD80/86-expressing macrophages is a driver of COVID-19 severity. In non-pathological conditions, the lungs are the tissue expressing the highest levels of *CD80* mRNA and the second tissue expressing more *CD86* mRNA (Supplementary Fig. S3A,B). This high basal expression of the key elements of the CD80/86 axis might reflect the need for a rapid activation of the immune system in an organ that is systematically exposed to multiple environmental cues^15^. However, this enhanced potential for T cell activation could become a highly detrimental factor in the presence of a rapidly propagating immune insult like SARS-CoV-2. A large population of CD80/86 APCs activated by a fast-spreading infection will lead to a higher activation of T cells which, in turn, will increase the activation proinflammatory cell programs, including IL6 production by lung macrophages^16^ (Fig. 8). This high signal amplification potential could therefore explain the sudden transition into the extremely high cytokine production stage observed during the cytokine storm of most COVID-19 fatalities^17^.

We have found that the treatment with abatacept significantly down-regulates myeloid leukocyte differentiation and macrophage activation at the systemic level. The hyperinflammatory stage in COVID-19 is characterized by the activation of myeloid cells, mostly monocyte-derived macrophages that massively infiltrate the lung^5^. Therefore, the specific anti-inflammatory effect of this therapy could be a useful property to avoid entering into the life-threatening massive cytokine release stage. Our results also show that treatment with abatacept downregulates the pathways associated with both the production of and the response to several key cytokines associated to the severe stage in COVID-19, including TNF^18^, IL1^19^, IL8^20^, IL7^18^ and IL6^18^. In particular, the downregulation by abatacept of systemic IL6 -the proinflammatory cytokine that has been shown to be predominant in patients progressing to severity^17^—was found to be highly significant. Our study suggests that IL6 production can also be effectively reduced by the upstream regulation of the immune system mediated by abatacept.

Our longitudinal analysis of the systemic response to abatacept showed that blocking of the CD80/86 axis leads to a strong downregulation of complement activation and B cell mediated immunity. Excessive complement activation has been associated to damage in the lungs of COVID-19 patients, and antibody therapies against elements of the complement system are currently being evaluated to treat severe cases^21^. An over-activation of the B cell mediated response could also be a harmful mechanism in severe COVID-19. During the SARS-CoV1 epidemic, deceased patients were found to have higher levels of neutralizing antibodies^22^, which raised the possibility of disease exacerbation through antibody-dependent enhancement (ADE)^23,24^. Experimental evidence with rhesus macaques infected with SARS-CoV-1 showed that IgG antibodies against the spike protein contributed to the massive influx of monocytes to the lung and subsequent severe injury^25^. The observed downregulation of the B cell immunity by abatacept might reduce the likelihood of low-affinity antibodies and the probability of ADE in patients^26^. To our knowledge, however, solid evidence that ADE contributes to COVID-19 severity has not been yet provided^27^.

A limitation of our study is that the longitudinal response to abatacept was measured in RA patients and not directly in COVID-19 patients. Therefore, our analysis of the systemic changes due to abatacept could have been confounded by the specific immune activation of RA. Like severe COVID-19^28^, however, RA is a disease where increased production of proinflammatory cytokines IL-6, IL-1β, and TNF is central to the pathological hyperinflammatory process^29^. Our longitudinal study design was key to capturing the effect and direction of abatacept-mediated regulation of these key pathways associated to severity. Nonetheless, a direct analysis of the transcriptional impact abatacept on SARS-CoV-2 infected patients or animal models was not evaluated. This is a limitation, because other aspects more specific to viral immune response will not have been represented. A direct analysis of the clinical and transcriptional impact abatacept on SARS-CoV-2 infected patients would be the ideal design to confirm our findings. In October 20th 2020, the US National Institutes of Health launched the ACTIV-1 IM protocol (#NCT04593940), a randomized clinical trial to evaluate immune modulators for the treatment of moderately or severely ill hospitalized COVID-19 patients. From a pool of more than 130 immune modulators, three agents, including abatacept, were selected to be evaluated in a randomized clinical trial with > 2000 COVID-19 patients admitted to the hospital. Our results show that there is potential for stratification of patients according to the gene signature at baseline. If the clinical trial shows a protective role for abatacept in severe COVID-19, our abatacept score could be tested for validation and be the basis of a biomarker profile to prioritize patients for preventative treatment.

There is increasing evidence that abatacept is an efficacious therapy to treat interstitial lung disease (ILD) in RA^30^. ILD is a comorbidity of RA that is characterized by the inflammation of the lung tissue and subsequent fibrotic development, which are also two canonical features of severe COVID-19. Treatment of this comorbidity with abatacept was motivated by the finding that blocking T cell co-stimulation is highly effective in a mouse model of hypersensitivity pneumonitis^31^. Much like in severe COVID-19, hypersensitivity pneumonitis is characterized by a massive influx of activated T cells in the lungs and a high expression of B7 (i.e. mouse CD80/CD86 molecules) by alveolar macrophages. Evidence from other diseases also support the role of the CD80/86 activation mechanism in excessive inflammatory responses in the lung^32,33^. Although the triggering immunogenic insults will be different in each disease, the importance of the CD80/86 axis in the immune response of the lungs might lead to a convergent hyperinflammatory response.

In support of the safety and utility of CD80/86 axis blocking to prevent severe COVID-19, there is substantial epidemiological evidence showing that abatacept does not increase the risk of serious infections^34–36^. While other immunosuppressive agents have been shown to increase this susceptibility to infections in rheumatic patients, abatacept seems to render the immune system fully capable of fighting off viral infections. In accordance with this previous evidence, in our abatacept-treated cohort of patients, we have found that basic antiviral response mechanisms like the type I interferon response were not significantly altered by the therapeutic blocking of the CD80/86 axis. Together, these observations support the safety of using this therapy to prevent the development of severe COVID-19.

An extraordinary international effort and unprecedented collaborations have recently led to the identification of vaccines for COVID-19 that appear to be safe and efficacious. Despite this formidable advance, there is a clear consensus that it is still critical to identify therapies that can reduce the high mortality rates of COVID-19 patients. The results of our study provide additional functional evidence that blocking of the CD80/86 axis could prevent reaching the hyperinflammatory stage observed in severe COVID-19. Abatacept has been used to treat RA for almost 15 years, and therefore there is a wide knowledge supporting the safety of this immunomodulatory drug. Taken together, our results support that abatacept should be considered a candidate therapy to prevent severe COVID-19.

## Methods

### Patients and samples

A total of n = 38 rheumatoid arthritis (RA) patients starting treatment with abatacept were recruited in the framework of the PACTABA project (BMS). All patients were treated with subcutaneous abatacept at the recommended weekly dosage of 125 mg. The PACTABA project is a multicentric Spanish pharmacogenomic study performed in a subset of patients from the ASCORE clinical trial (Bristol-Myers Squibb, ClinicalTrials.gov Identifier: NCT02090556). This clinical trial was an observational and prospective study designed to estimate the retention rate of subcutaneous abatacept over 24 months in the routine clinical practice of RA patients^37^. The study was approved by the Vall d’Hebron University Hospital institutional review board and informed consent was obtained in all cases. All research was performed in accordance with the Declaration of Helsinki, and informed consent was obtained from all participants. Supplementary Table S10 shows the clinical features of the RA patient cohort.

Whole blood samples were obtained from all patients at the beginning of the therapy with abatacept and at week 12. Blood was collected using RNA-stabilizing PaxGene tubes (PreAnalytiX, Switzerland), to preserve total RNA integrity from the time of venipuncture.

### RNA-seq data generation and preprocessing

Total RNA was extracted using the PaxGene blood isolation kit (Qiagen). All samples had a RIN index > 7 and were included for RNA-seq analysis. RNA-seq libraries were performed using with the KAPA RNA HyperPrep Kit, with RiboErase (HMR) Globin (globin and rRNA depletion protocol) for Illumina sequencing platforms. In order to improve the accuracy of the gene expression quantification, we added Unique Molecule Identifiers (UMIs). This was performed during the library construction, in which xGen UDI-UMI Adapters (IDT) were ligated after the second strand synthesis. These full-length adapters include P5 and P7 sequences that are compatible with Illumina sequencing platforms. Sequencing was performed using the NovaSeq platform (Illumina) at average of 250 paired-end reads. A total of 3,376,481,954 sequencing reads were obtained in the two conditions (weeks 0 and 12). FASTQ files were subsequently aligned to the GRCh37 human reference genome assembly using STAR 2.7.6a^38^. The global alignment ratio (mean of all samples) was 95.2% (Supplementary Fig. S4 shows the read mapping distribution in all the samples and Supplementary Table S11 the per-sample QC). After alignment, we performed deduplication of the PCR duplicates using UMItools^39^ to mark duplicates and samtools^40^ to remove them. Gene-level read counts of the deduplicated bam files were obtained with *featureCounts*^41^ for gene annotations downloaded from Ensembl Genes GRCh37v75. This resulted in a total of 3,212,779,029 (95.15%) of overall reads mapping as pairs to annotated genes. The RA longitudinal dataset is available through the NCBI Gene Expression Omnibus database with id GSE151161.

To characterize the gene expression profile associated with COVID-19, we used RNA-seq data from two COVID-19 cohorts. The first cohort^13^ (early cohort) consists on the transcriptional profile on peripheral blood mononuclear cells (PBMCs) from three COVID-19-positive patients and three healthy controls from Wuhan, China and made available in March 2020. The raw sequencing data was processed as described by the authors. The second cohort^14^ (late cohort) consists on the whole blood RNA-seq profile from n = 100 COVID-19-positive patients admitted to the Albany Medical Center (NY, USA). Severe patients were defined as COVID-19 patients having less than 25 hospital free days (HFD45 < 25) in a 45 day follow-up.

### Differential expression and gene set enrichment analysis

To determine the gene expression changes induced at a systemic level by the treatment with abatacept, we performed a longitudinal analysis using the linear regression framework implemented in *limma*^42^. First, we selected genes with a minimal evidence of gene expression in blood, defined as > 0.6 counts per million in at least 20 samples. TMM-normalized values were then log-transformed using *voom*^43^ and a multivariate linear model was fitted accounting for repeated measures per individual (i.e. baseline and week 12 measures), and also adjusting for sex, age and batch effects (i.e. sequencing library plate number). In the early COVID-19 cohort we identified an unequal percentage of granulocytes between cases and controls (Supplementary Fig. S5). For this reason, for analysis involving this cohort, adjustment was also performed for granulocyte proportions (estimated using DeconCell^44^). A total of n = 44 genes were found to be differentially expressed (FDR < 0.05) without granulocyte-adjustment, and n = 101 when adjusting. The genes differentially expressed between COVID-19 patients and healthy controls were determined using *edgeR*^45^ with adjustment for granulocyte percentage (sex and age information were not available in this dataset). Genes with low expression values (defined as < 2 counts per million in at least 2 samples) were previously filtered out. In the late COVID-19 cohort, we used the RSEM counts provided by the authors^14^ and differential expression analysis was performed like in the abatacept dataset. A total of n = 1065 genes were found to be differentially expressed (FDR < 0.05) between severe and mild COVID-19 patients.

Testing for up- or down-regulation of biological processes due to the treatment with abatacept and by COVID-19 was performed using the Gene Set Enrichment Analysis (GSEA)^46^ method. The GSEA is a commonly used method to identify coordinated differences in expression in functionally-related sets of genes. In this method, a normalized enrichment score (NES) is calculated for each pathway. The NES measures if the genes in a particular pathway are over-represented at the top or at the bottom of a ranked list of genes. In our analysis, we ranked genes based on the corresponding differential expression results. In order to be able to test for antagonism, the directionality of the differential gene expression was preserved by multiplying the -log (P-value) of the differential expression by the sign of the fold change, as described previously^47^. This resulted in a ranked list of n = 18,370 genes, n = 14,829 genes, n = 12,389 genes, in the Abatacept, early COVID-19 and late COVID-19 datasets respectively, that are used as input in the GSEA analysis. For each biological pathway, P-values for association were calculated using the adaptive Monte-Carlo method implemented in the *fgsea* package with n = 1e6 permutations. GO terms of size < 10 or > 300 where excluded, and the GSEA parameter value was kept to 1 (default). Multiple test p-value adjustment was performed using Benjamini and Hochberg false discovery rate (FDR) method^48^.

### Analysis of the antagonism of abatacept to COVID-19

To determine the level of antagonism induced by abatacept to COVID-19 pathology in the two COVID-19 cohorts we used two alternative approaches. In the first approach we analyzed exclusively biological processes that have been consistently associated with COVID-19 disease pathology and, principally, with disease severity. After manually identifying the most cited studies and more replicated evidence, we identified a total of 22 relevant processes and its representing GO term. Supplementary Table S1 describes the most relevant bibliographical evidences for each process. In order to avoid a potential bias in considering pathways with a very similar gene composition, we measured the overlap between each pair of pathways using the Jaccard Index (Supplementary Fig. S6). We found a low degree of overlap in most of the pathway annotations, ruling out the bias of redundant process annotation. The list of pathology-associated processes can be divided according to the two immune response stages of COVID-19: (i) anti-viral response mechanisms (i.e. first stage of COVID-19, n = 6)^49^, and (ii) hyperinflammatory process and disease severity (i.e. second stage of COVID-19, n = 16)^3,19,21,50^. The change in activity of these processes induced by abatacept was subsequently tested using the GSEA approach. Antagonistic processes were determined as those were the pathway was significantly modified by abatacept (P-value < 0.05) and in the opposite direction to that described for COVID-19 patients.

In the second approach, we determined the level of antagonism of abatacept to COVID-19 using an agnostic analysis, and therefore not limited to the current knowledge of the disease. In this approach, we determined the biological pathways associated to COVID-19 as those differentially activated by the disease at the transcriptional level using the case–control RNA-seq dataset. We next determined all the biological pathways differentially activated by treatment with abatacept. In both cases, pathway annotation was defined using the GO biological process annotation. Pathways with less than 10 genes or more than 300 genes were excluded. The FDR method was applied to correct for multiple testing; an FDR < 0.05 was considered significant. Since GO terms that are functionally close can have very similar gene composition, we used the Jaccard index to identify redundant annotations. For this objective, a distance matrix between all pairs of BPs was built using 1 − *Jaccard Index*. Using this Jaccard distance matrix, clusters of redundant BPs were then defined using hierarchical clustering and cutting at height *h* = 0.6. For each cluster, the GO term with the most significant association was selected for downstream analysis, leading to the selection of n = 6187 GO terms. Similar to the previous approach, antagonistic processes were determined as those that are significantly modified by abatacept (FDR < 0.05) and in the opposite direction to that found in the COVID-19 case–control group (FDR < 0.05). In order to test for the significance of the observed antagonistic processes a binomial test was used. Details of this analysis are included in the supplementary methods.

### Survival analysis

In order to investigate the potential benefits of abatacept to prevent severity, we developed a transcriptional-based score that captures the similarity to abatacept-induced signature. The methodology to build this score (abatacept score, ABS) is described in the supplementary material. Using the score distribution in the complete patient cohort (COVID-19-positive and negative patients, Supplementary Fig. S7), we selected the patients showing the more extreme ABS values for downstream analysis.

To evaluate the association between the ABS patient categorization with severity, we performed two survival analyses. In both analyses, Cox proportional hazards models by Kaplan–Meier estimation were carried out using outcomes of interest. In the first analysis, Cox proportional hazards were estimated using days without mechanical ventilation, considering intubation (yes or no) as censoring status. In the second analysis, a Cox proportional hazards model was conducted to evaluate the differences between the two ABS groups with regards to the number of days spent in the hospital. As censorship status, we used the categorization of patients into severe and mild COVID-19 groups based on the HFD-45 described in the original manuscript^14^ (i.e. < 25 days as severe and ≥ 25 as mild patients).

### Single cell RNA-seq analysis of COVID-19 BALF cells

In order to corroborate the relevance of the CD80/86 signaling axis in COVID-19, we analyzed single cell RNA-seq data generate from the lungs, the target tissue of severity. For this objective we analyzed a single cell RNA-seq recently generated from bronchoalveolar lavage fluid (BALF) cells from COVID-19 patients and healthy controls (GEO database accession GSE145926)^10,11^. Single-cell RNA-seq raw data from BALF samples was generated using the 10× Genomics platform and included 9 COVID-19 patients (n = 6 severe and n = 3 mild). The raw data was downloaded and processed using Seurat (v3)^51,52^ and scTransform^53^. scTransform performs normalization and variance stabilization of single-cell RNA-seq data using regularized negative binomial regression, which has shown to effectively reduce the effect of technical variation, without affecting biological heterogeneity. This dataset initially consisted of a total of 62,460 cells. Cells with < 200 or > 6000 unique feature counts, > 10% mitochondrial counts or < 1000 UMIs were subsequently filtered out. After this quality control, a total 54,420 cells (87.1%) were available for downstream analyses. Samples were log-normalized and scaled for the number of genes, number of UMIs and percentage of mitochondrial reads. Cell type clustering was performed using the *FindClusters* function from Seurat using the default resolution (0.8). This clustering method uses a shared nearest neighbour (SNN) modularity optimization-based clustering algorithm to identify cell clusters of cells based on their PCs. The number of PCs used for each clustering round (k = 50) was estimated by the elbow criterion in the PCA scree plot (Supplementary Fig. S8). Using this approach, we identified a total of 34 cell clusters (Fig. 5A,B). The cell clusters aggregated into four major regions representing (i) T and NK cells (n = 8014 cells, 14.7%, clusters 9, 11, 5, 25 and 18), (ii) two regions of FABP4^+^ alveolar macrophage cells (n = 17,399 cells, 32%, clusters 1, 2, 3, 30, 7, 32 and 12), and (iii) a large cluster aggregate of active, *IL6*-expressing macrophages both from FABP4^+^ alveolar and monocyte-derived FCN1^+^ macrophages (n = 24,742 cells, 45.5%, clusters 6, 20, 13, 22, 0, 8, 4, 15, 10, 16, 14, 26). As described by the original study, FABP4^+^ alveolar macrophages show a marked transition to FCN1^+^ monocyte-derived macrophages from healthy to infected and as COVID-19 severity increases (data not shown). Cluster 5 aggregated markers of different types of CD4+ T cells including *CCR7* (naïve TCD4), *IL2RA* (Treg), *FOXP3* (Treg), *IL7R* (naive), *LTB* (naive), *CXCL13* (T peripheral helper) (Fig. 6C). This cluster also was characterized by the upregulation of CTLA4, the protein that is recombined in abatacept, and also a marker that is highly upregulated in active T cells^54^. Consequently, this cell cluster was classified as activated CD4+ T cells. COVID-19 patients had a much higher number of CD80^+^ and CD86^+^ macrophages compared to controls (19.6% vs 1.7% and 45.4% vs 26.5%, respectively, P < 1e−16 in both comparisons) (Fig. 6A). IL6-expressing macrophages, were present almost exclusively in COVID-19 patients (Fig. 6B). Association between the key cell types (CD80^+^, CD86^+^ macrophages, active T cells and IL-6 expressing macrophages) was performed using linear regression. Log-transformed cell counts were normally distributed (i.e., Shapiro–Wilk test P > 0.05) and were used in this analysis. To account for potential confounding due to differences in total cell numbers, these were included as a covariate in the regression model.

## Supplementary Information

Supplementary Information 1.

Supplementary Information 2.

## Data Availability

The code used to generate the results and figures of this study is openly available at https://github.com/Rheumatology-Research-Group/COVID-19_Abatacept.
