# Supplementary material for: Targeting of the CD80/86 proinflammatory axis as a therapeutic strategy to prevent severe COVID-19
Source: Sci Rep. 2021 Jun 1;11:11462. doi: 10.1038/s41598-021-90797-0 (PMC8169841; doi:10.1038/s41598-021-90797-0)
Supplement: Supplementary file 1 — Supplementary Information 1. [file 41598_2021_90797_MOESM1_ESM.docx]

**Targeting of the CD80/86 proinflammatory axis as a therapeutic strategy to prevent severe COVID-19**

Antonio Julià^1, *^, Irene Bonafonte^1^, Antonio Gómez^1^, María López-Lasanta^1^, Mireia López-Corbeto^1^, Sergio H. Martínez-Mateu^1^, Jordi Lladós^1^, Iván Rodríguez-Nunez^2^, Richard M. Myers^2^, Sara Marsal^1, *^

**Supplementary Table 1. List of biological processes associated with COVID-19 severity and associated studies**

| Biological category | Biological process | Implication in COVID-19 | References |
| --- | --- | --- | --- |
| Viral entry into cell | endosomal transport | Mechanism of viral entry into cell | ^1^,^2^,^3^,^4^,^5^,^6^ |
| Virus sensing | toll−like receptor signaling pathway | Prevents cell infection. Evaded by coronaviruses | ^1^,^3^,^7^,^8^ |
| Virus sensing | cytoplasmic pattern recognition receptor signaling pathway in response to virus | Prevents cell infection. Evaded by coronaviruses | ^1^,^9^,^10^,^11^ |
| Virus sensing | type I interferon signaling pathway | Unclear. Impaired in severe COVID-19 | ^12^,^13^,^14^,^15^,^16^,^11^ |
| Natural killer mediated immunity | natural killer cell chemotaxis | Over-activation in COVID-19 | ^17^,^18^,^19^ |
| Natural killer mediated immunity | natural killer cell mediated cytotoxicity | Down-regulated in COVID-19 | ^20^,^21^,^19^ |
| Blood coagulation | regulation of blood coagulation | Up-regulated in severe COVID19 | ^22^,^23^,^15^ |
| T cells | T cell mediated immunity | Blood lymphopenia, altered function, activated and exhausted in severe. Increased in lung. | ^24^,^25^,^25^,^20^,^26^,^8^,^27^,^28^,^29^,^30^,^17^,^31^ |
| T cells | T cell cytokine production | Up-regulated in severe COVID19 | ^24^,^25^,^30^,^17^,^31^ |
| T cell interaction with myeloid cells | antigen processing and presentation | Up-regulated in severe COVID19 | ^32^,^33^,^34^ |
| T cell interaction with myeloid cells | response to interferon−gamma | Up-regulated in severe COVID19 | ^35^,^24^,^36^,^15^ |
| T cell interaction with myeloid cells | cellular response to tumor necrosis factor | Up-regulated in severe COVID19 | ^24^,^15^,^37^ |
| Myeloid cell activation | myeloid leukocyte differentiation | Up-regulated in severe COVID19 | ^24^,^25^,^33^,^8^,^38^,^17^ |
| Myeloid cell activation | macrophage activation | Up-regulated in severe COVID19 | ^24^,^25^,^33^,^8^,^17^,^23^ |
| Cytokine production | interleukin−1 production | Up-regulated in severe COVID19 | ^35^,^8^,^31^,^15^ |
| Cytokine production | interleukin−6 production | Up-regulated in severe COVID19 | ^39^,^24^,^26^,^14^,^33^,^38^,^27^,^23^,^36^ |
| Cytokine production | tumor necrosis factor production | Up-regulated in severe COVID19 | ^35^,^39^,^24^,^26^,^14^,^33^,^40^,^23^ |
| Cytokine production | interleukin−8 production | Up-regulated in severe COVID19 | ^35^,^39^,^26^,^23^ |
| Cytokine production | response to interleukin−7 | Up-regulated in severe COVID19 | ^35^,^24^,^41^ |
| Cytokine production | interleukin−10 production | Up-regulated in severe COVID19 | ^35^,^39^,^26^,^27^,^36^ |
| Complement pathway | complement activation | Up-regulated in severe COVID19 | ^42^,^43^,^44^,^45^ |
| Ig production by B cells | B cell mediated immunity | Up-regulated in severe COVID19 | ^14,6^,^4,7^,^30^ |

**Supplementary Figure 1. A**


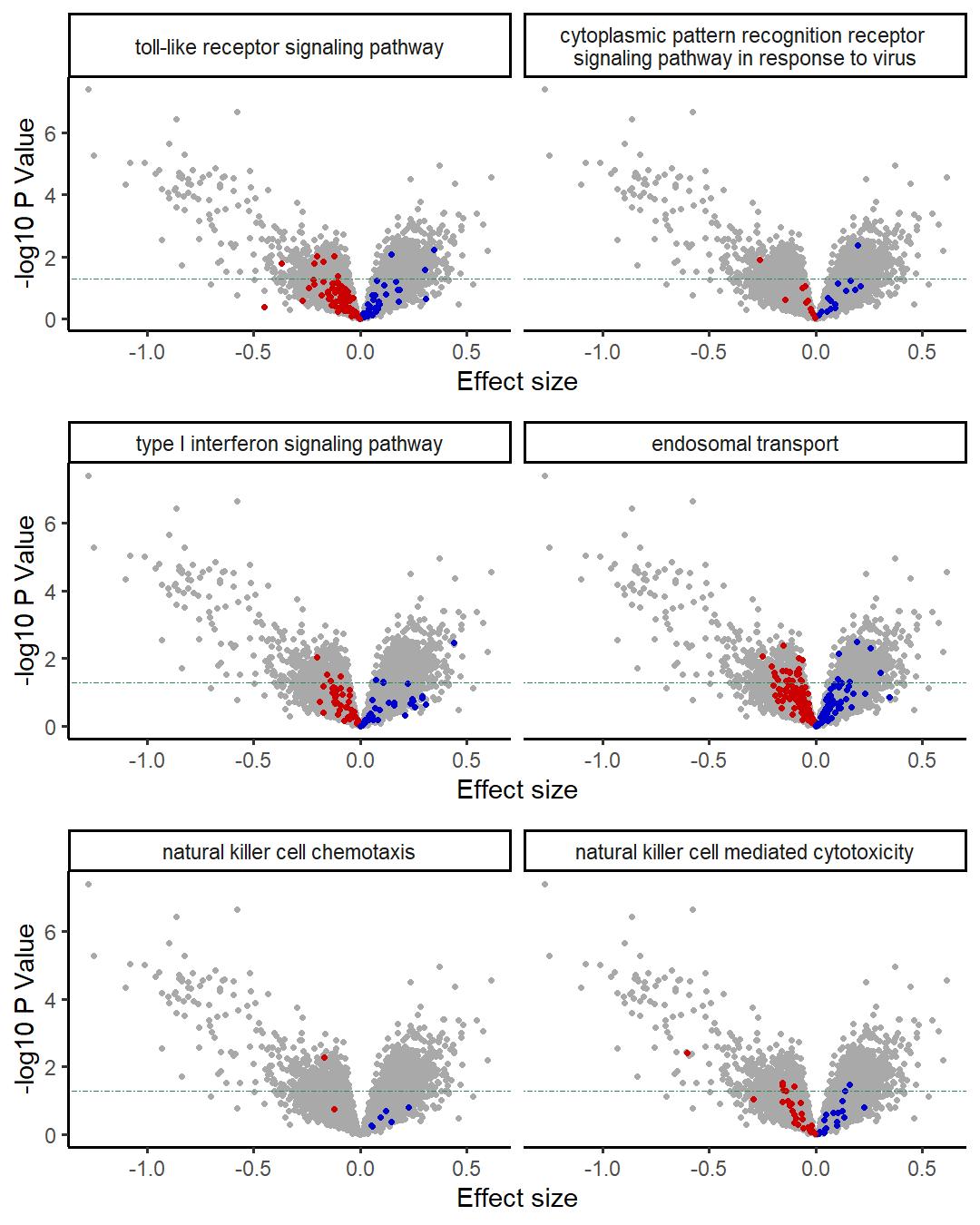


**Supplementary Figure 1. B**


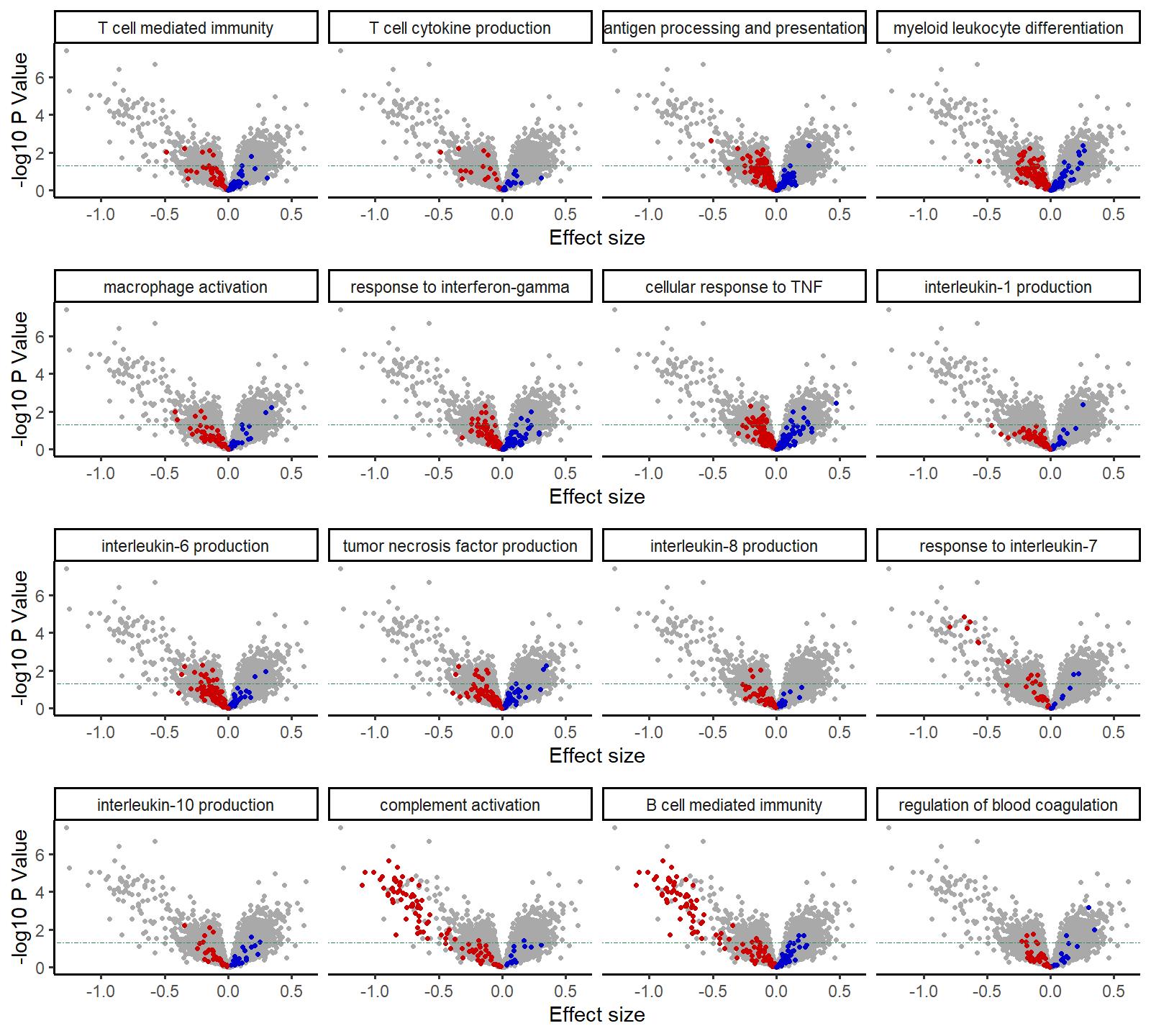


**Supplementary Figure 1. Gene-level differential expression induced by abatacept for the 22 processes characteristic of COVID-19 pathology. A.** Processes associated to the response to viral infection (first stage of COVID-19). **B.** Immune processes associated with COVID-19 hyperinflammation (second stage of COVID-19). Volcano-plots showing the differential expression results for all genes after 12 weeks of treatment with abatacept. The statistical significance of each gene (-log10(P value), y-axis) is plotted against the effect size (log fold change, x-axis). The genes composing the particular biological process are highlighted in color, with red indicated the genes downregulated by abatacept and blue indicating the genes upregulated by the drug.

**A**

**
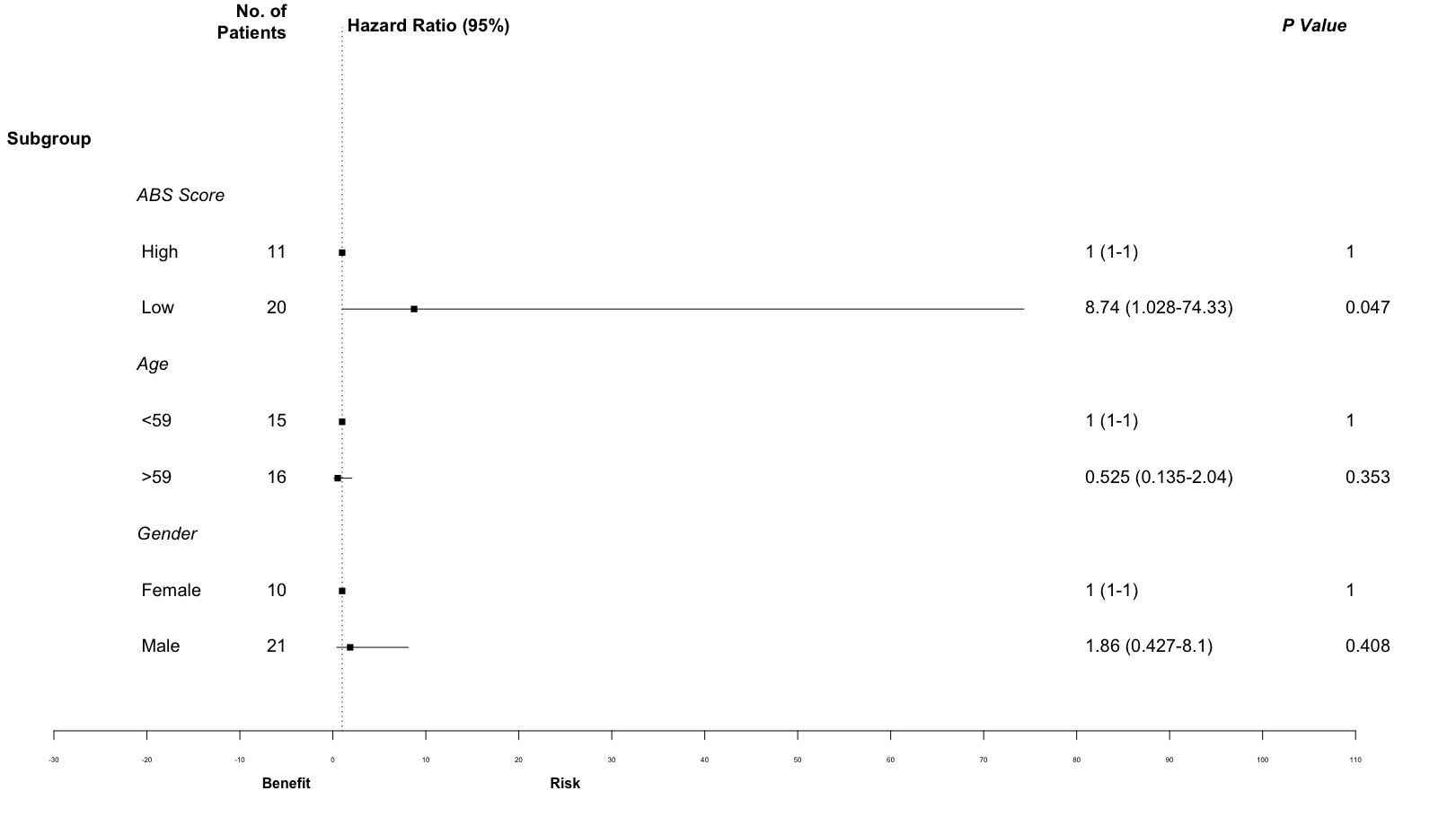
**

**B**

**
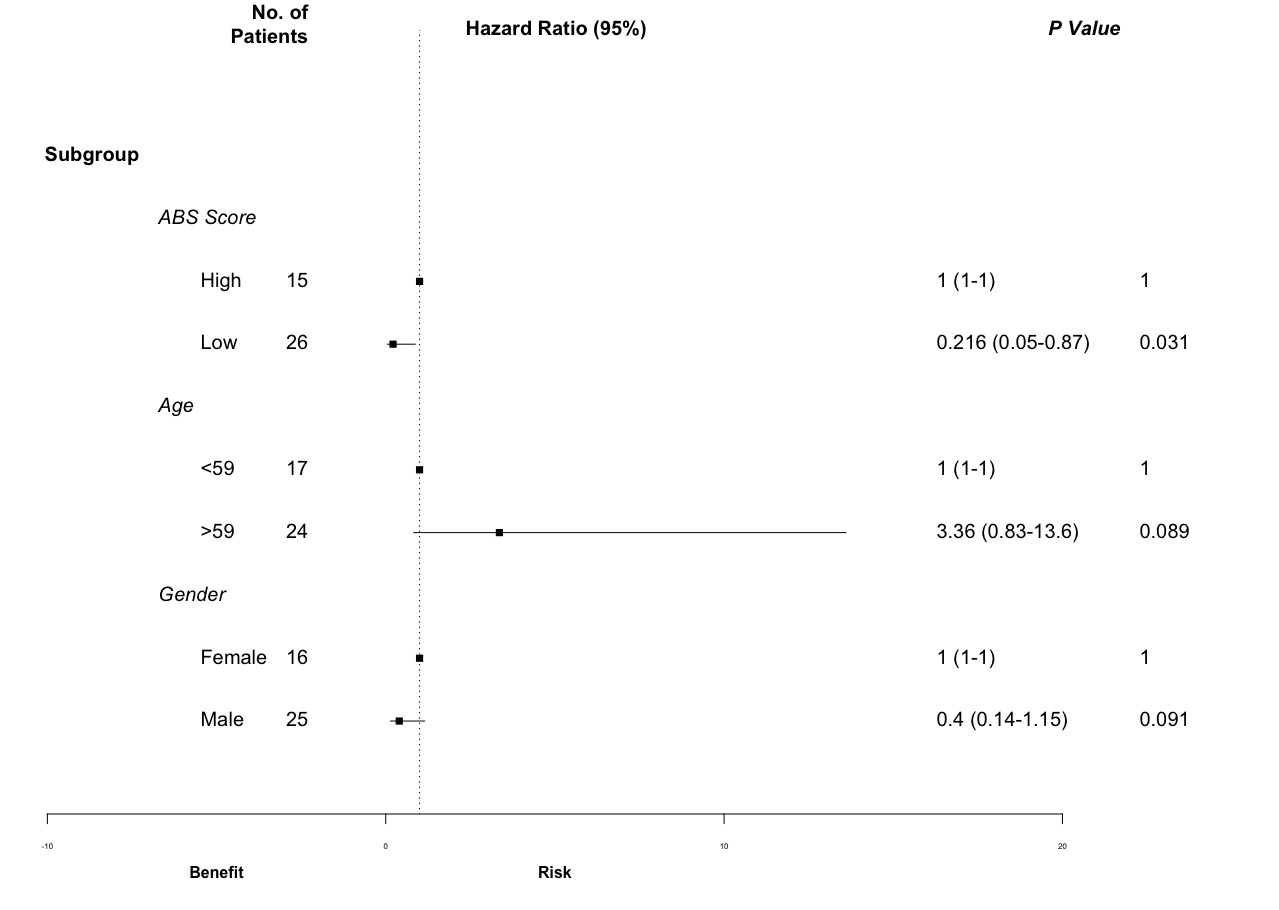
**

**Supplementary Figure 2. Hazard ratio forest plots. A.** Hazard ratios estimated according to the number of days free of mechanical ventilation. The hazard ratio for a primary outcome event in the ABS-Low group was 8.74 (95% confidence interval [CI], 1.03 to 74.33; P=0.047, log-rank test). **B.** Forest-plot of the differences according to the Cox proportional hazards model in the analysis of days of hospitalization. In this analysis, ABS-Low patients are found to spend more days at the hospital, and this difference is statistically significant (P = 0.031 log-rank test). Note that, according to the outcome, the hazard is here annotated as “benefit” and, therefore, it does not have the literal meaning.

**Supplementary Figure 3. A**


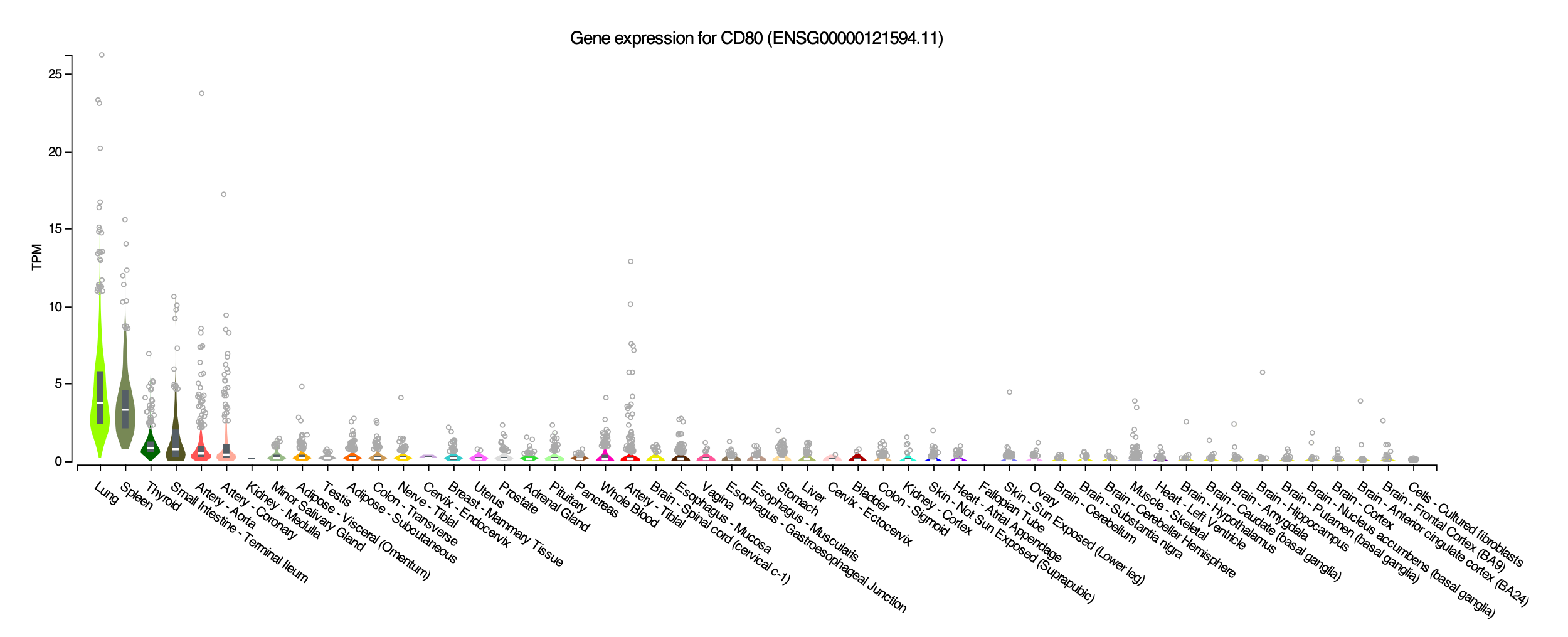


**Supplementary Figure 3. B**


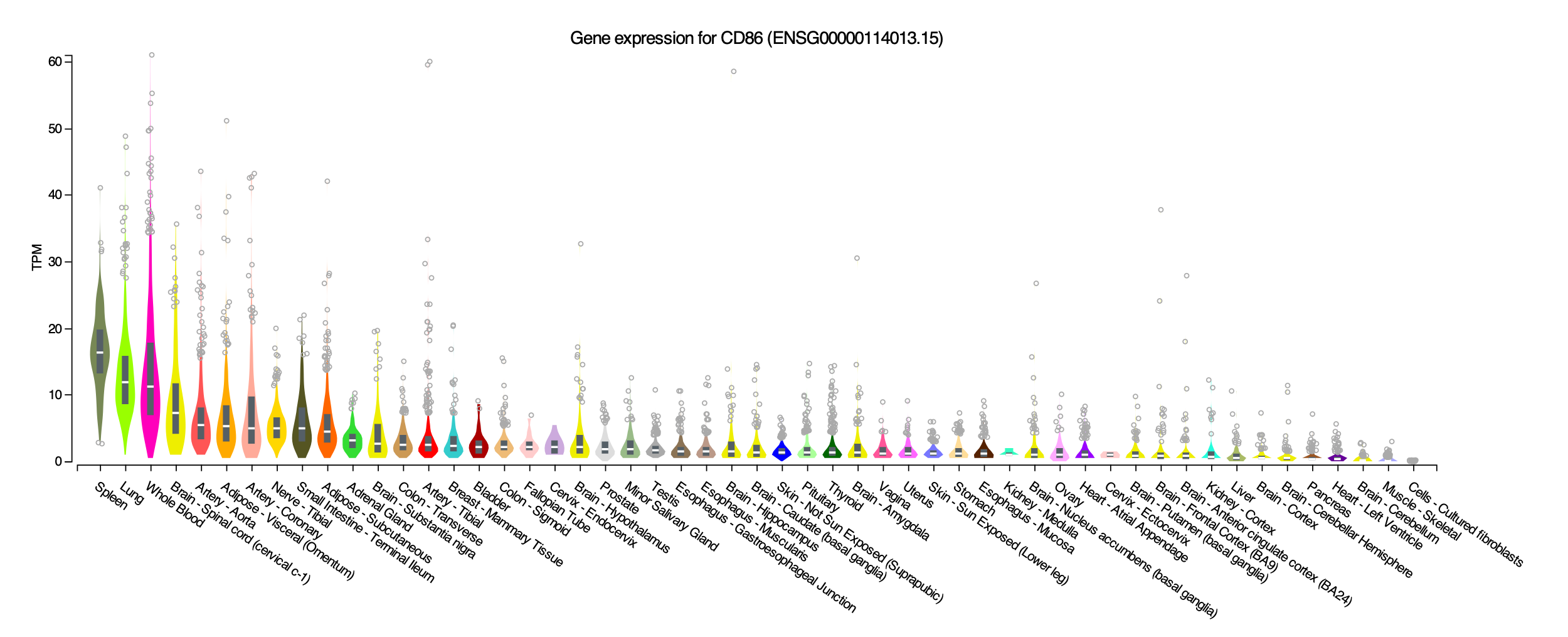


**Supplementary Figure 3. Tissue-level gene expression distribution of CD80 and CD86.** Sorted distribution of gene expression levels for the two genes across 53 human tissues from nearly 1,000 individuals are shown (GTex database, version 8). **A.** Tissue expression of CD80 shows that the lungs (light green) are the tissue expressing the higher mRNA levels of this gene. **B.** Tissue expression of CD86 shows that the lungs (light green) are the second tissue with a higher expression of this gene.

**
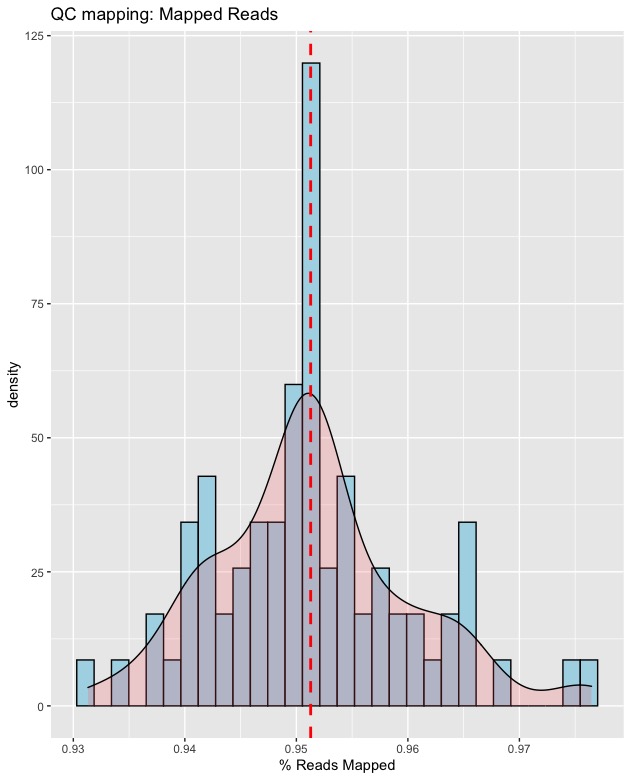
**

**Supplementary Figure 4. Blood RNA-seq read mapping percentage, Abatacept cohort.** Density plot showing the distribution of mapped reads among the 76 longitudinal blood RNA samples from RA patients sequenced in this study. The vertical dashed red line indicates the mean (95.2%).

**Supplementary Figure 5. Evidence of granulocyte imbalance between the cases and controls in the early COVID-19 dataset.** Cell type deconvolution into the major blood cell subsets, showed one sample with a larger contribution of neutrophil RNA. In order to correct for this potential confounder, we used the granulocyte (neutrophil) percentage as a covariate in the differential expression analysis. N1-3: control samples; P1-P3: patient samples.

**
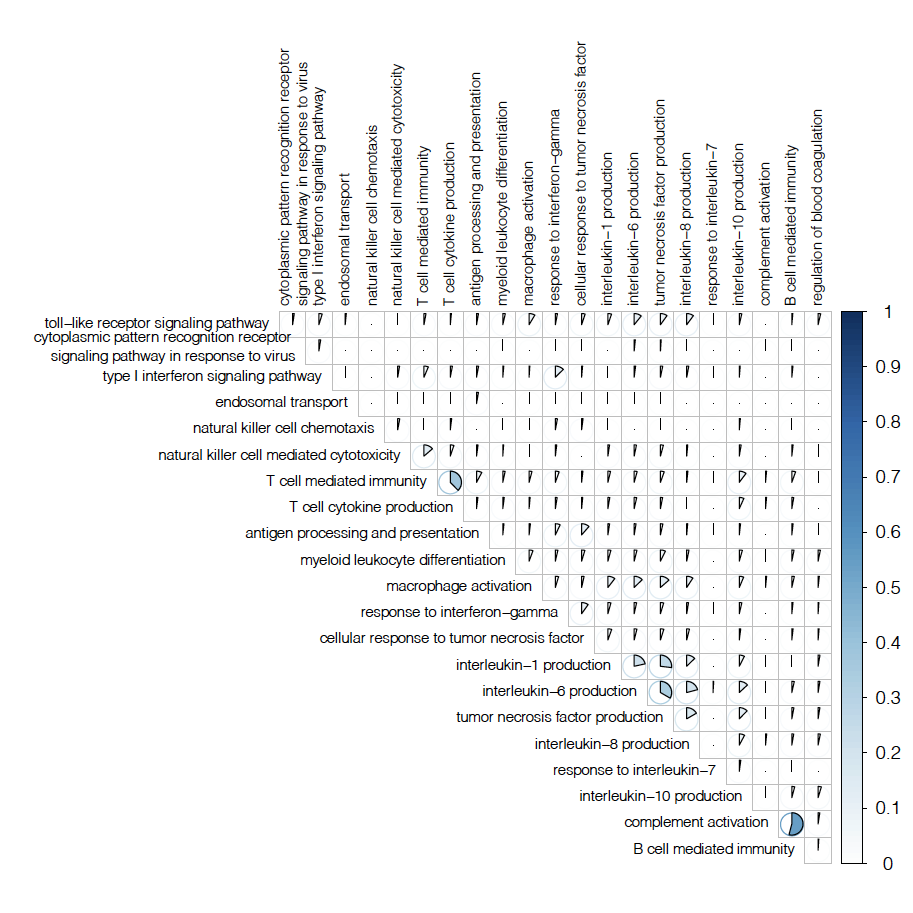
**

**Supplementary Figure 6. Gene content overlap between the 22 biological processes associated with COVID-19 pathology.** Pairwise comparison of the degree of overlap between the biological processes using 1-Jaccard index measure. Using this measure, pathways having an identical gene composition would have a score of 1, while pathways not sharing a single gene would have a score of 0. Each pie chart indicates the overlap score both by the gradient color and the size of the pie section. Despite describing processes of the immune-response domain, the overlap is low between these pathways. Only one pair of annotations show a score > 0.5, involving B cell mediated immunity and complement activation.

**A**

**B
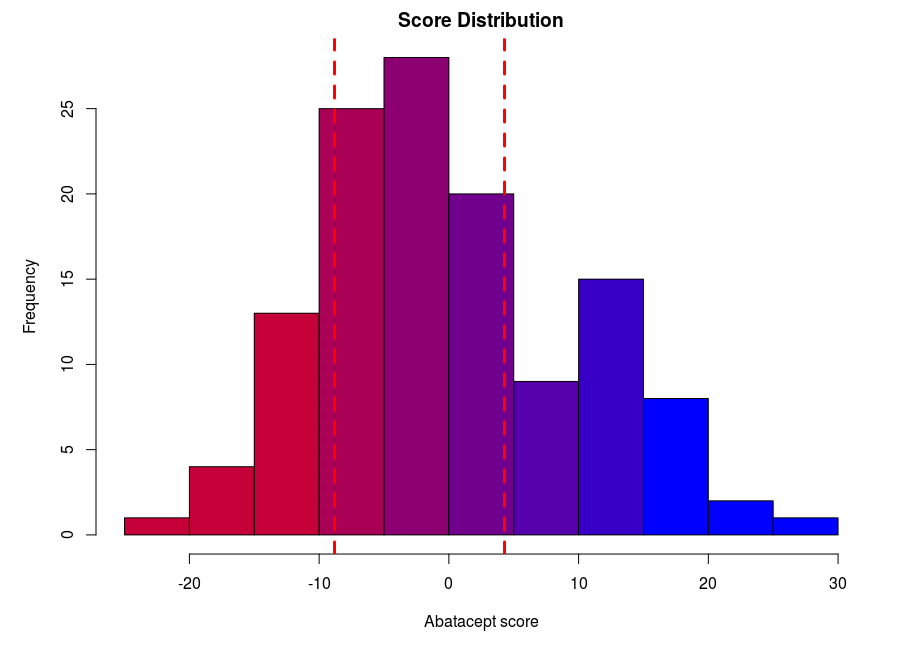
**

**
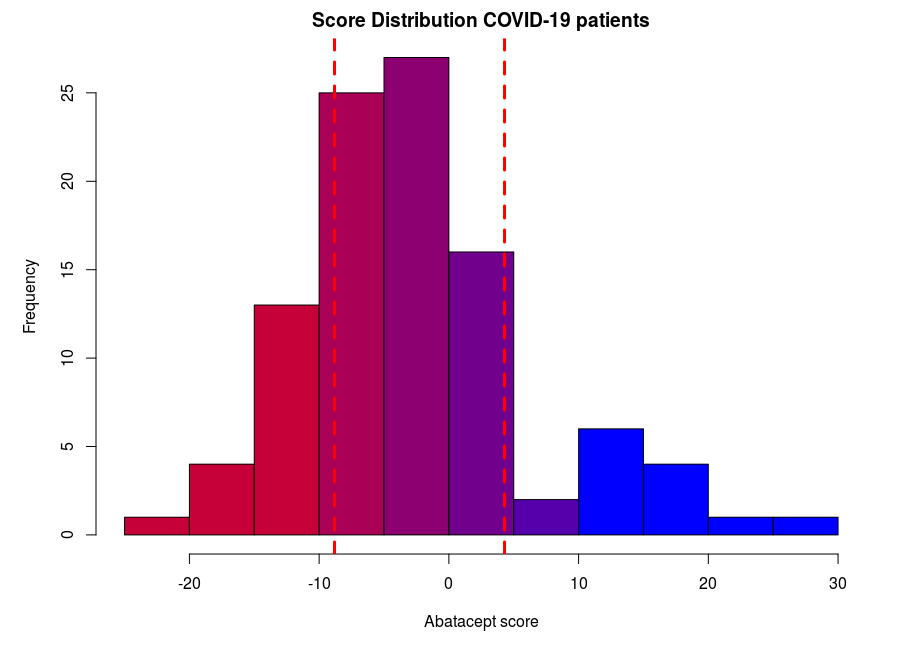
**

**C**

**
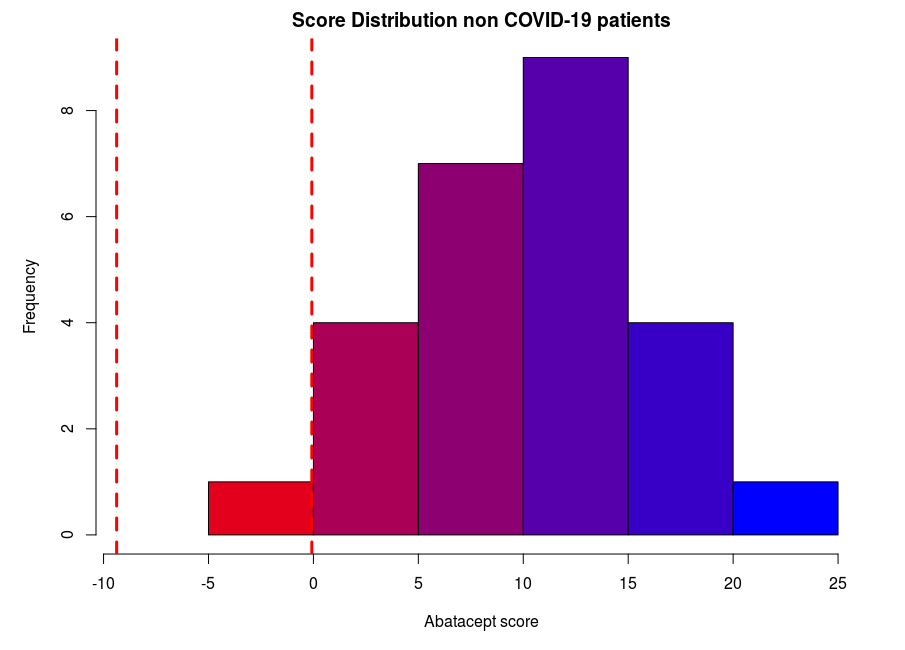
**

**Supplementary Figure 7. Abatacept score distribution. A.** Histogram of the ABS score in the n=100 COVID-positive and n=26 COVID-negative patients from the late COVID-19 cohort. The dashed vertical lines indicate the cut-offs used to select the patients with more extreme signature values for the downstream analyses. ABS-High and ABS-Low categories were based on the quantile distribution of the score, using the 20^th^ and 70^th^ percentiles (dashed lines) as cutoffs, respectively. **B.** Histogram of the ABS score in the COVID19-positive patients only. **C.** Histogram of the ABS score in the COVID19-negative patients only.

**
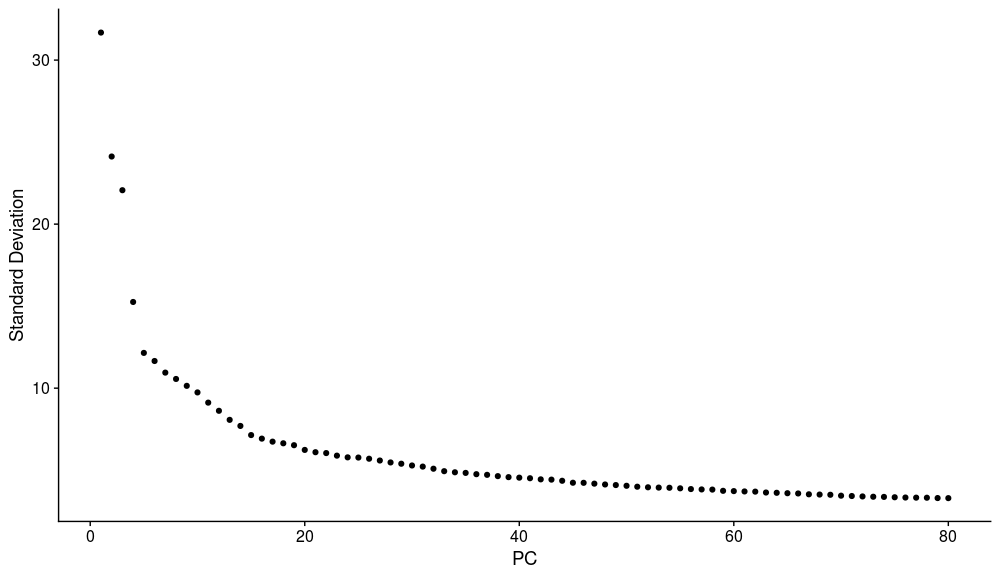
**

**Supplementary Figure 8. Scree plot of the COVID-19 BALF scRNA-seq dataset.** Standard deviations of the first n=80 principal components of the case-control scRNA-seq dataset bronchoalveolar cell dataset analyzed in this study.

**Supplementary Table 10. Main epidemiological and clinical features of the abatacept-treated RA cohort.**

| **Clinical variable** | **Summary** |
| --- | --- |
| Age (years), mean ± SD | 59.4 ± 12.9 |
| Female, n (%) | 28 (73.6%) |
| Disease duration (years), median (IQR) | 8.5 (9.5) |
| DAS28 week 0, median (IQR) | 5.59 (1.67) |
| DAS28 week12, median (IQR) | 4.42 (2.26) |
| Anti-CCP Positive, n (%) | 33 (86.8%) |
| Prior biologic treatments (number), mean ± SD | 1.0 ± 1.22 |

SD: standard deviation; IQR: interquartile range; DAS28: disease activity score for 28 joints ^48^; anti-CCP: anti-cyclic citrullinated peptide antibodies.

**Supplementary Methods: Statistical test for antagonism**

To test the significance of the observed proportion of antagonism between the pathways associated in the COVID19 datasets vs the abatacept-response dataset the binomial model was used. To do this, we defined the Expected probability (**p0**) as:

$$p0=\left( nCOVID.up/ntrials \right)\times\left( nABA.down/ntrials \right)+(nCOVID.down/ntrials) \times(nABA.up/ntrials)$$

where *ntrials* corresponds to the total number of analyzed BPs (after excluding reduntant BPs), *nCOVID.up* the number of BPs significantly up-regulated in the COVID-19 datasets (adjusted *P* < 0.05, NES > 0), *nCOVID.down* the number of BPs significantly down-regulated in the COVID-19 datasets (adjusted *P* < 0.05, NES < 0), *nABA.up* the number of BPs significantly up-regulated in the abatacept cohort (adjusted *P* < 0.05, NES > 0), and *nABA.down* the number of BPs significantly down-regulated in the abatacept cohort (adjusted *P* < 0.05, NES < 0).

We define the number of successes (*nSuccesses*) as the number of BPs that are significantly regulated in opposite direction in the COVID-19 and abatacept cohorts. Using a binomial test, the empirical proportion of antagonistic cases, $p=\left( nSuccesses/ntrials \right)$ is compared to the expected probability *p0* (using the *binom.test* function from the R *stats* package) to determine if such proportion of successes is higher than expected by chance.

**Supplementary Methods: Abatacept response similarity score**

In order to determine the level of similarity of the transcriptional profile of patients from the late COVID-19 cohort^49^ to that of the signature of response to abatacept we build a score (ABS). To build the score we used the set of genes showing differential expression (FDR <0.05) between baseline and week 12 and with detectable gene expression counts in the large COVID-19 datasets (n=15 genes). These genes include: *CDC20, TOX2, IGHA1, IGHG2, KIFC1, IGLC2, TYMS, RRM2, IGLC3, BIRC5, IGHG4, IGHA2, GTSE1, HJURP, IGHG1*.

The expression values of the signature genes were then standardized, using mean centering and scaling to unit standard deviation. Then, for each COVID patient, a linear combination of the standardized expression values of the signature genes was performed using the coefficients of the differential expression analysis. Formally, the ABS for patient *i* was computed as follows:

$${ABS}_{i}= {SE}_{i}\left( G_{1} \right)\times logFC\left( G_{1} \right)+{SE}_{i}\left( G_{2} \right)\times logFC\left( G_{2} \right)+\cdots+{SE}_{i}(G_{n})\times logFC\left( G_{n} \right)$$

Where *G_1_, G_2_, …, G_n_* are the signature genes, SE_i_(G_j_) is the standardized expression value of the j-th gene in the i-th patient and logFC(G_j_) is the coefficient of the j-th gene in the differential expression analysis performed in the abatacept cohort. This way, high-scoring patients (ABS-High) will have a transcriptomic profile in blood that resembles that of an abatacept-treated patient, while low-scoring patients (ABS-Low) will be more similar to abatacept-untreated individuals.

**References**

1. Vabret, N. *et al.* Immunology of COVID-19: current state of the science. *Immunity* (2020).

2. Hu, T. Y., Frieman, M. & Wolfram, J. Insights from nanomedicine into chloroquine efficacy against COVID-19. *Nat. Nanotechnol.* **15**, 247–249 (2020).

3. Zizzo, G. & Cohen, P. L. Imperfect storm: is interleukin-33 the Achilles heel of COVID-19? *Lancet Rheumatol.* (2020).

4. Hirano, T. & Murakami, M. COVID-19: A new virus, but a familiar receptor and cytokine release syndrome. *Immunity* (2020).

5. Hoffmann, M. *et al.* SARS-CoV-2 cell entry depends on ACE2 and TMPRSS2 and is blocked by a clinically proven protease inhibitor. *Cell* (2020).

6. Krammer, F. SARS-CoV-2 vaccines in development. *Nature* **586**, 516–527 (2020).

7. Iwasaki, A. & Yang, Y. The potential danger of suboptimal antibody responses in COVID-19. *Nat. Rev. Immunol.* 1–3 (2020).

8. Ong, E. Z. *et al.* A dynamic immune response shapes COVID-19 progression. *Cell Host Microbe* (2020).

9. Hackbart, M., Deng, X. & Baker, S. C. Coronavirus endoribonuclease targets viral polyuridine sequences to evade activating host sensors. *Proc. Natl. Acad. Sci.* **117**, 8094–8103 (2020).

10. Zhang, Y. *et al.* β-arrestin 2 as an activator of cGAS-STING signaling and target of viral immune evasion. *Nat. Commun.* **11**, 1–15 (2020).

11. Xia, H. *et al.* Evasion of type I interferon by SARS-CoV-2. *Cell Rep.* **33**, 108234 (2020).

12. Zhou, Z. *et al.* Overly exuberant innate immune response to SARS-CoV-2 infection. (2020).

13. Shi, Y. *et al.* COVID-19 infection: the perspectives on immune responses. (2020).

14. Hadjadj, J. *et al.* Impaired type I interferon activity and exacerbated inflammatory responses in severe Covid-19 patients. *MedRxiv* (2020).

15. Lucas, C. *et al.* Longitudinal analyses reveal immunological misfiring in severe COVID-19. *Nature* **584**, 463–469 (2020).

16. Lee, J. S. & Shin, E.-C. The type I interferon response in COVID-19: implications for treatment. *Nat. Rev. Immunol.* **20**, 585–586 (2020).

17. Liao, M. *et al.* The landscape of lung bronchoalveolar immune cells in COVID-19 revealed by single-cell RNA sequencing. *medRxiv* (2020).

18. Guo, C. *et al.* Tocilizumab treatment in severe COVID-19 patients attenuates the inflammatory storm incited by monocyte centric immune interactions revealed by single-cell analysis. *bioRxiv* 2020.04.08.029769 (2020). doi:10.1101/2020.04.08.029769

19. Maucourant, C. *et al.* Natural killer cell immunotypes related to COVID-19 disease severity. *Sci. Immunol.* **5**, (2020).

20. Zheng, M. *et al.* Functional exhaustion of antiviral lymphocytes in COVID-19 patients. *Cell. Mol. Immunol.* 1–3 (2020).

21. Wilk, A. J. *et al.* A single-cell atlas of the peripheral immune response to severe COVID-19. *medRxiv* (2020).

22. Tang, N., Li, D., Wang, X. & Sun, Z. Abnormal coagulation parameters are associated with poor prognosis in patients with novel coronavirus pneumonia. *J. Thromb. Haemost.* (2020).

23. Zhang, W. *et al.* The use of anti-inflammatory drugs in the treatment of people with severe coronavirus disease 2019 (COVID-19): The experience of clinical immunologists from China. *Clin. Immunol.* 108393 (2020).

24. Merad, M. & Martin, J. C. Pathological inflammation in patients with COVID-19: a key role for monocytes and macrophages. *Nat. Rev. Immunol.* 1–8 (2020).

25. Zhou, Y. *et al.* Aberrant pathogenic GM-CSF+ T cells and inflammatory CD14+ CD16+ monocytes in severe pulmonary syndrome patients of a new coronavirus. *BioRxiv* (2020).

26. Qin, C. *et al.* Dysregulation of immune response in patients with COVID-19 in Wuhan, China. *Clin. Infect. Dis.* (2020).

27. Chen, G. *et al.* Clinical and immunological features of severe and moderate coronavirus disease 2019. *J. Clin. Invest.* **130**, (2020).

28. Diao, B. *et al.* Reduction and functional exhaustion of T cells in patients with coronavirus disease 2019 (COVID-19). *Front. Immunol.* **11**, 827 (2020).

29. Tan, L. *et al.* Lymphopenia predicts disease severity of COVID-19: a descriptive and predictive study. *Signal Transduct. Target. Ther.* **5**, 1–3 (2020).

30. Anft, M. *et al.* A possible role of immunopathogenesis in COVID-19 progression. *medRxiv* (2020).

31. Xu, Z. *et al.* Pathological findings of COVID-19 associated with acute respiratory distress syndrome. *Lancet Respir. Med.* **8**, 420–422 (2020).

32. Grifoni Alba; Weiskopf, D. R. S. I. . M. J. D. J. M. . M. C. R. R. S. A. ; S. A. P. L. J. R. S. . M. D. de S. A. M. . F. A. ; C. A. G. J. A. . Targets of T cell responses to SARS-CoV-2 coronavirus in humans with COVID-19 disease and unexposed individuals. *Cell* (2020).

33. Giamarellos-Bourboulis, E. J. *et al.* Complex immune dysregulation in COVID-19 patients with severe respiratory failure. *Cell Host Microbe* (2020).

34. Swadling, L. & Maini, M. K. T cells in COVID-19—united in diversity. *Nat. Immunol.* **21**, 1307–1308 (2020).

35. Huang, C. *et al.* Clinical features of patients infected with 2019 novel coronavirus in Wuhan, China. *Lancet* **395**, 497–506 (2020).

36. Liu, J. *et al.* Longitudinal characteristics of lymphocyte responses and cytokine profiles in the peripheral blood of SARS-CoV-2 infected patients. *EBioMedicine* 102763 (2020).

37. Buszko, M. *et al.* The dynamic changes in cytokine responses in COVID-19: a snapshot of the current state of knowledge. (2020).

38. Zhang, D. *et al.* COVID-19 infection induces readily detectable morphological and inflammation-related phenotypic changes in peripheral blood monocytes, the severity of which correlate with patient outcome. *medRxiv* (2020).

39. Gong, J. *et al.* Correlation analysis between disease severity and inflammation-related parameters in patients with COVID-19 pneumonia. *MedRxiv* (2020).

40. Liao, Y., Smyth, G. K. & Shi, W. featureCounts: an efficient general purpose program for assigning sequence reads to genomic features. *Bioinformatics* **30**, 923–930 (2014).

41. Mehta, P. *et al.* COVID-19: consider cytokine storm syndromes and immunosuppression. *Lancet* **395**, 1033–1034 (2020).

42. Gao, T. *et al.* Highly pathogenic coronavirus N protein aggravates lung injury by MASP-2-mediated complement over-activation. *MedRxiv* (2020).

43. Holter, J. C. *et al.* Systemic complement activation is associated with respiratory failure in COVID-19 hospitalized patients. *Proc. Natl. Acad. Sci.* **117**, 25018–25025 (2020).

44. Risitano, A. M. *et al.* Complement as a target in COVID-19? *Nat. Rev. Immunol.* **20**, 343–344 (2020).

45. Ramlall, V. *et al.* Immune complement and coagulation dysfunction in adverse outcomes of SARS-CoV-2 infection. *Nat. Med.* **26**, 1609–1615 (2020).

46. Okba, N. M. A. *et al.* Severe Acute Respiratory Syndrome Coronavirus 2-Specific Antibody Responses in Coronavirus Disease 2019 Patients. *Emerg. Infect. Dis.* **26**, (2020).

47. Zhang, L. *et al.* Antibody responses against SARS coronavirus are correlated with disease outcome of infected individuals. *J. Med. Virol.* **78**, 1–8 (2006).

48. Van Riel, P. L. & Renskers, L. The Disease Activity Score (DAS) and the Disease Activity Score using 28 joint counts (DAS28) in the management of rheumatoid arthritis. *Clin Exp Rheumatol* **34**, S40--S44 (2016).

49. Overmyer, K. A. *et al.* Large-scale multi-omic analysis of COVID-19 severity. *Cell Syst.* (2020).
